# Mechanistic insights into excitonic and electrostatic stimulation of cells by photovoltaic substrates/nanocrystals and through light polarization modulation

**DOI:** 10.1371/journal.pone.0335978

**Published:** 2025-11-07

**Authors:** Mohammad Mohammadiaria, Daniel L. Rathbun, Moses Kamita, Shashi Bhushan Srivastava

**Affiliations:** 1 Independent Researcher, Pavia, Italy; 2 Henry Ford Health + Michigan State University Health Sciences, Detroit, Michigan, United States of America; 3 Department of Hematology/Oncology, Henry Ford Health, Detroit, Michigan, United States of America; Nazarbayev University, KAZAKHSTAN

## Abstract

Photoelectrical stimulation of cells and neural modulation via the separation of photo-induced electrical charges in photocapacitor structures have proven effective and biocompatible for therapeutic applications, such as retinal prostheses. Recent advances in photovoltaic materials and device architectures, particularly the use of pixelated photoelectrodes, have enabled high-resolution modulation of neuronal transmembrane potentials. Upon illumination, photo-induced dipoles and excitons in semiconductor layers generate localized electric fields that interact with the cell membrane to trigger stimulation. Polarization-modulated light dynamically alters the orientation of these dipoles, modulating field orientation and enhancing light–matter coupling at the membrane interface. This effect is especially pronounced in anisotropic media or aqueous environments, where polarization control enables deeper, more focused light penetration. Our framework combines (1) a photocapacitive mechanism that displaces charge across the cell membrane through excitonic microdomain redistribution in the photovoltaic hybrid and (2) an electrostatic force from photo-induced dipoles near the cell. These effects are embedded in an equivalent-circuit model that links optical inputs (intensity and polarization) to the device’s open-circuit voltage (V_OC_) and photocurrent (I_ph_), and subsequently to the resulting membrane potential (V_m_). Using the PCE12:ITIC-based solar cell platform, we experimentally demonstrate polarization-dependent modulation of photovoltage and photocurrent, and directly correlate these effects with intracellular calcium dynamics. Calcium imaging of hippocampal neurons revealed robust, stimulus-locked ΔF/F₀ transients on PCE12:ITIC substrates under light stimulation, in contrast to minimal responses on control ITO films, confirming that polarization-modulated excitonic processes drive physiologically relevant changes in neuronal signaling. Moreover, we highlight how dipole–membrane coupling provides a conceptual and functional link between neuromodulation and quantum logic systems, especially when realized through nanocrystal-based harmonic oscillators. InP-ZnO nanoclusters exhibit selective responses to left circularly polarized (LCP) light, offering pixel-wise selectivity for color-encoded retinal stimulation. Bioinspired anisotropic quantum dot arrays, modeled after polarization-sensitive ommatidia in bee eyes, enable spatially selective neuromodulation and programmable bio-optoelectronic interfaces.

## Introduction

**Fig 1:**
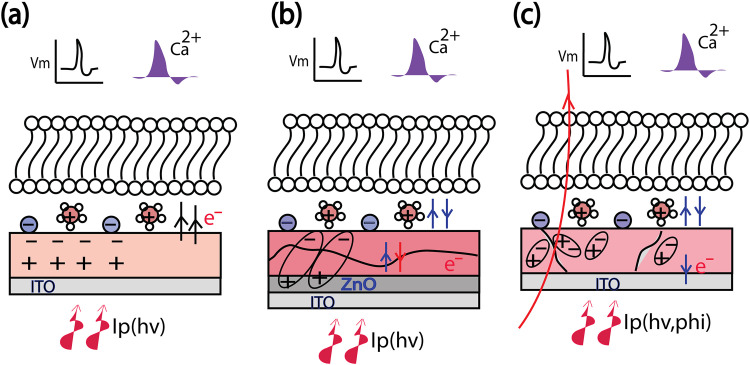
Different cellular mechanisms based on photofaradaic, photocapacitive, and excitonic stimulations. (a) Photofaradaic-capacitive stimulation [8,11], (b) Photocapacitive stimulation of cells with engineered band-alignment in a photovoltaic cell [3,17], and (c) Polarized light modulation inducing electric field redistribution and facilitating capacitive and pixel-wise stimulation based on microdomains in a hybrid organic solar cell without band alignment engineering.

Photoelectrical stimulation has been widely applied as an alternative to optogenetics [1] for cellular modulation [2–4]. There are different forms of photoelectrochemical stimulation, including photo-induced charge separation or dipole formation [5,6], photocapacitive, photofaradaic [3,7,8], and photochemical processes [9,10]. The latter three mechanisms respectively modulate membrane capacitance, membrane potential, or intracellular calcium, ultimately leading to cellular stimulation. One of the safer stimulation mechanisms is through photocapacitive currents generated by photovoltaic substrates. In general, photo-induced dipoles in a semiconductor can generate an external electric field or photopotential at the interface, arising from charge separation and redistribution within the semiconductor and the surrounding medium [11]. Through engineering of the electronic structure in a semiconductor device we can control charge transfer and electrical field displacements at the biointerface. There are various photovoltaic and semiconductor options, including organic hybrids [12,13], Perovskite [14,15], and quantum dots [2] that have been used to generate photo-induced dipoles in both solar cells and neural interfaces. Such photovoltaic stimulation holds significant promise for restoring vision in cases of blindness, particularly through retinal implants [16–18]. These implants and associated biointerfaces can function either as pixelated, closed-loop systems [18–23] or as freestanding, untethered configurations [24]. A second type of photoelectrical stimulation is through photo-Faradaic ionic currents or through the accumulation of superoxides [2,3], which can lead to harmful alterations of intracellular reactive oxygen species (ROS) or intracellular calcium levels. For instance, potassium voltage-gated channels in neurons can be opened by selective electrochemical currents, such as those generated by hydrogen peroxide [25]. In contrast, photo-capacitive stimulation facilitates the photo-induced electric field displacement (through photo-induced electrical dipoles in the photocapacitor) and the redistribution of electrical charges across the cell membrane. This type of electrical stimulation minimizes harmful photoelectrochemical and photothermal effects. Moreover, light polarization modulation can also result in the generation of a photo-potential, enabling neural stimulation by electric field displacement (or inducing electrostatic force on a patch of the cell membrane) without the need for a closed-loop current, thanks to excitonic cell stimulation. Light polarization modulation offers advantages such as deeper light penetration in the medium and pixel-wise control of cellular activation [26,27]. Fig 1 compares the different types of photoelectrical stimulation with the excitonic method that is introduced for the first time in this paper.

Leveraging polarization-sensitive materials, including aligned nanorods, anisotropic quantum dots [28], or liquid-crystalline polymers [29,30], may enable spatially selective or orientation-specific stimulation. Furthermore, integrating polarization as a gating mechanism could enhance the specificity of photovoltaic or photocapacitive platforms, opening pathways for bidirectional interfaces that both actuate and read out cellular alignment and high spatiotemporal resolution for in vivo cellular stimulation as an advanced alternative to normal polymeric nanoparticles [31]. The orientation of the photo-induced dipoles has been shown to control optoelectronic functionality of photovoltaic junctions, such as perovskite junctions, through different incident polarized s and p lights [32]. While *p-*polarized transition dipoles cause high charge dissociation and collection via the anode and cathode electrodes, resulting in increased photo-current generation, *s*-polarized transition dipoles lead to high photoluminescence. Similarly, in neural interfaces, the band energy alignment and the direction of photo-induced dipoles could induce different cell transmembrane potentials. The photo-induced dipole (PID) effect in quantum dot sensitized solar cells (QDSSCs) produces a high open-circuit voltage due to a negative shift in the energy band of titanium oxide (TiO2) [10]. Kazes et al confirmed the PID effect in a metal oxide organic solar cell structure that enabled a high open-voltage circuit and photo-capacitive stimulation of cells [33].

**Fig 2:**
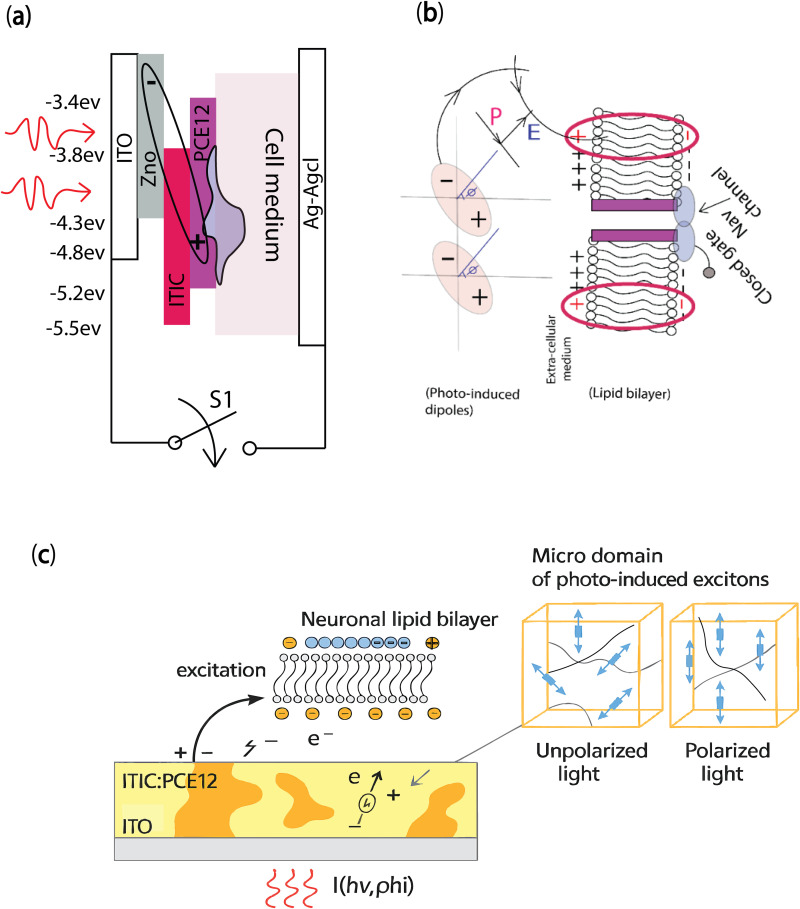
(a) A cartoon of the photo-induced dipole (demonstrated based on the energy band diagram) in a bulk organic photo-capacitor structure and a grown neuron on the surface of the bio-interface. (b) Dipole-dipole interaction between photo-induced dipoles and the electrical charges across the cell lipid bilayer (without considering the effect of the electrical double layer). (c) Unaligned and aligned dipole microdomains induced by polarized light in a photovoltaic organic hybrid system. Each microdomain represents localized excitonic regions formed upon photoexcitation, with alignment enhanced under polarization, illustrating the anisotropic photoresponse of the hybrid material.

Based on the energy band alignment in a metal-oxide semiconductor junction, the direction of the internal photo-induced dipoles and the capacitive behaviour of the junction could be controlled. Fig 2(a) shows a photo-induced dipole in the ITO/ poly[(2,6-(4,8-bis(5-(2-ethylhexyl)thiophen-2-yl)-benzo[1,2-b:4,5-b’]dithiphene))-alt-(5,5-(1’,3’-di-2-thienyl-5’,7’-bis(32-ethylhexyl)benzo[1’,2’-c:4’,5’-c’]dithiophene-4,8-dione))] (PBDB-T or PCE12): 3,9-bis(2-methylene-(3-(1,1-dicyanomethylene)indanone))-5,5,11,11-tetrakis(4-hexylpheny)-dithieno[2,3,-d:2’,3’-d’]-s-indaceno[1,2-b:5,6-b’]dithiophene (ITIC) photovoltaic junction under light excitation. Fig 2(b) presents the dipole-dipole interaction between the photo-induced dipoles in a metal oxide-semiconductor junction and the dipoles of the lipid bilayer. This, in fact, happens when the dipolar orientation in the organic bulk heterojunction photosensitive layer alters the polarization of the photopotential at the interface fast enough that no electrical charge transfer or ionic reaction occurs at the surface of the photocapacitor and the ionic medium. We consider a theoretical dipolar interaction in comparison to whole cell recording measurements from an experiment involving photostimulation of cells on Indium tin oxide/Titanium oxide/Indium phosphate QDs. In fact, from the electrical circuit model, an external electric field is generated by the photo-electrode to alter the membrane potential but at the same time, a dipolar interaction could induce an electrostatic force on the lipid bilayer. In this context, the non-fullerene acceptor ITIC, when blended with donor polymers such as PCE10 or PEC12, offers significant advantages over traditional fullerene-based systems such as P3HT:PCBM [34,35].

These blends exhibit stronger absorption in the visible spectrum, enhanced excitonic dissociation, and improved charge transport [36]. Critically, the anisotropic molecular packing and tunable energy levels of ITIC-based systems make them particularly responsive to polarization-modulated light, enabling efficient excitonic field alignment and stronger photocurrent generation under dynamic optical stimuli. This property is highly desirable for bio-interfacing applications such as retinal stimulation, where polarization sensitivity and high charge separation efficiency directly enhance neuromodulation performance. For instance, in this paper, we used PCE12:ITIC for excitonic stimulation without the band alignment engineering of the interface. PCE12:ITIC and P3HT:PCBM differ significantly in their excitonic behavior and electronic properties in organic solar cells. PCE12:ITIC blends typically exhibit stronger intramolecular charge transfer and more delocalized excitons due to the non-fullerene acceptor (ITIC), leading to reduced exciton binding energy and more efficient charge separation. We observed that PEC12:ITIC blends exhibit significantly reduced recombination losses and enhanced excitonic behavior compared to P3HT:PCBM. This can be attributed to the more delocalized excitons and stronger intramolecular charge transfer in PEC12:ITIC, resulting in more efficient charge separation and reduced exciton binding energy [37]. In contrast, P3HT:PCBM systems exhibit a higher exciton binding energy, resulting in more localized excitons that require stronger electric fields for dissociation. Furthermore, PCE12:ITIC systems often form more continuous donor–acceptor networks with favorable domain purity and phase separation, which improves charge transport and collection. These properties contribute to PCE12:ITIC devices generally achieving higher open-circuit voltages (V_oc_) compared to P3HT:PCBM. We observed enhanced performance with PCE12:ITIC blends under polarized light modulation, likely due to their improved molecular ordering, anisotropic domain alignment, and lower exciton binding energy. These properties facilitate more efficient dipole-field coupling and directional charge separation, making PCE12:ITIC particularly responsive to polarization-encoded optical stimulation compared to P3HT:PCBM systems.

Moreover, polarization modulation has facilitated the production of specific holographic patterns of light for optogenetics, as well as in three dimensions [38,39]. Likewise, with polarization modulation, different patterns of photocurrents or local field potentials could be generated on the surface of the photo-capacitors coupled to a metasurface. In this study, we investigate the polarization modulation for generating different photovoltages and photocurrents, both with and without quantum dots. In addition, a mathematical and electrical model has been introduced for excitonic stimulation of cells based on the photo-induced dipoles in a photo-electrode and the cell membrane for the substrates without a metasurface. This model gives more insights into how the photo-induced cell membrane potential can be modulated through the light polarization effect. In the following sections, we first explore excitonic cellular stimulation driven by polarized light in bulk heterojunction solar cell architectures. We then examine distinct mechanisms of photo-induced stimulation at the light–semiconductor interface, including dipole-mediated interactions (induced electric fields), and photo-generated charge accumulation, each contributing uniquely to cellular modulation.

## Results & discussion

### The mechanism of excitonic stimulation by photo-induced dipoles in a bulk photovoltaic substrate

In general, photo-induced dipoles create extracellular electric fields that can interact directly with the cell’s lipid bilayer (dipole-dipole interaction) or with extracellular ions (dipole-charge interaction). Figs 2(a) and 2(b) show the schematic and energy band diagram of a photo-capacitor, as well as the photo-induced dipoles under light excitation. As demonstrated recently [40], high spatial resolution in organic optobioelectronic devices is critically governed by the properties of photo-induced dipoles formed within nanoscale microdomains of organic semiconductor blends. Recently, in bulk heterojunction solar cells such as PCE12:ITIC-based systems, light absorption generates excitons, bound electron-hole pairs, that dissociate at donor–acceptor interfaces, producing localized photoinduced dipoles within phase-separated microdomains (Fig 2(c)). These dipoles arise from spatially confined charge separation in the organic hybrid matrix, where nanoscale morphology dictates both exciton diffusion and interfacial dynamics. The collective behavior of these synthetic dipoles contributes to the internal electric field, influencing both the open-circuit voltage (V_oc_) and photocurrent (I_ph_). Significantly, the polarization state of incident laser light can modulate the orientation of dipoles within these microdomains, effectively rotating their alignment and tuning the anisotropy of the generated field (Fig 2(c) inset). Polarized excitation can consequently enhance directional charge separation or, conversely, suppress interfacial charge transfer by orienting dipoles away from donor–acceptor pathways. This optical control enables the system to transition from Faradaic (charge-transferring) to capacitive behavior, reducing current flow while maintaining field-induced stimulation. When interfaced with biological cells, this polarization-driven modulation of synthetic dipole fields enables tunable, non-invasive control of the membrane potential, offering a fundamentally novel and biocompatible approach to neuromodulation through light, without the need for direct ionic or molecular delivery.

Further, the direction and amplitude of photoinduced dipoles are controlled by incident light intensity and polarization. The fundamental equations (equation 1–3) are used to determine the mechanism operating in the hypothesis, establishing a mathematical relation between photovoltaic parameters and light polarization. Equation 1 describes the photo-induced dipoles in which the electrons and holes have different masses of m_n_ and m_h_ in different materials in a bulk heterojunction. Equation 2 describes the photo-induced potential as a function of the input light intensity, the intrinsic properties of the semiconductor, and the polarization of the incident light. Equation 3 describes the surface potential drop at the surface due to the double-layer capacitance at the interface with the water. A linear polarization of the light leads to an electrical field displacement in the x and y planes as a Cosine wave, like what has been reported for Si polarizer detectors [15]. At a specific input light intensity, the output photovoltage is saturated and reaches a maximum level. Subsequently, the photo-induced dipole reaches its highest magnitude, as described in the open-circuit voltage in equation 2. A harmonic oscillator can be generated by modulating light polarization between 90° and 270°. In this way, an oscillating electric dipole can be produced to stimulate cells at a neuronal interface.


P(I, θ, x, y,t)=p=q.d=Q.d.cos θt (1)

where I is the light intensity amplitude, θ the polarization angle, Q the photoinduced charge, d the dipole distance, x and y the coordinates of illumination, and t the time.


$$q\;\left(E_{C}-E_{HOMO}\right).\text{cos}\;\theta =q.V_{OC}.\text{cos}\;\theta =q.V_{S}.\text{cos}\;\theta$$ (2)

Where the q is the electrical charges, E_C_ is the conduction energy band, E_HOMO_ is the HOMO level, and θ is the polarization angle that could be linear.


$$V_{S}\;(\theta ,\;r,t)=P.E.cos{\;\theta }_{t}=\frac{\text{1}}{\text{4}\pi \varepsilon_{\text{0}}}.\frac{p.r.cos\theta t}{R^{\text{2}}}$$ (3)

In which θ is the polarization angle, r is the radius from the photo-induced dipole, and t is the time.

#### Open-Loop condition.

In open-loop conditions, a minimal electrochemical reaction, the surface potential can be determined by the open circuit voltage of the photovoltaic device while using an ordinary incident light. However, the alternation of light polarization causes local field potentials, as described in Equation 2. In addition, a uniform illumination to a photovoltaic substrate (bulk model) causes an output photovoltage that can be calculated as equation 3, where the open-circuit voltage is obtained by the photo-induced charges and the energy differences. The photo-induced surface potential can open voltage-gated ion channels by altering the cell membrane potential or through the electrostatic force resulting from the dipolar interaction between the photo-induced dipoles and the lipid bilayer.

Further the change in polarization angle using polarizer can lead to to time-dependent fucntionality of charges. Hence, the polarization can be rewritten as a time-dependent dipole as follows.


P(t)=Q(t)·d·cos(θ(t)) (4)

Where, photogenerated charge accumulation with saturation can be represented as photovoltaic internal parameters as follows:


$$Q(t)=\frac{\eta_{q}G_{ph}(t)\tau_{life}}{\text{1}+G_{ph}(t)\tau_{life}/N_{max}}$$ (5)

Where η_q_ is quantum efficiency, G_ph_(t) is photon generation rate (e.g., light intensity × absorption coefficient), τ_life_ is carrier lifetime, N_max_: maximum charge density (saturation limit), d is effective dipole length, and θ(t) is angle between dipole and external field (e.g., light polarization).

#### Closed-Loop condition.

If there is a short-circuit loop from the substrate and the cell medium, the membrane potential could be displaced by the changes in the photocapacitor’s capacitance because of changing the orientation of the photo-induced dipoles through light polarization modulation as well. Figure S1 in S3 File in supplementary information shows the schematic, and the electrical circuit model of a neuron coupled to a photo-capacitor in a closed-loop condition. Under light excitation, a neuron is electrically stimulated through the electric field displacement at the surface of a bio-interface due to the photo-induced dipoles in the photo-capacitor. At the same time, the intensity controls the amplitude of the photo-induced dipole; the polarization of the incident light can alter the direction of the photo-induced dipole, the internal electric field, the capacitance of the photo-capacitor, and the photocurrent under light excitation. The effective electric field generated by the light excitation in a photocapacitor could be obtained from the following equation:


$$E_{effective}=E-E_{polarization}=\frac{\sigma }{k\varepsilon_{\text{0}}}$$ (6)

Where, the factor, $k=\frac{\sigma_{m}}{\varepsilon_{\text{0}}}\propto P$, and photocapacitance, $C=\frac{k\varepsilon_{\text{0}}A}{d}$. Therefore,


$${\Delta C}_{interface}=\;\propto \Delta P$$ (7)

Considering we have a linear polarization; k is altered with a cosine function similar to Equation 1. The factor k is affected directly by the photo-induced dipoles in a semiconductor under the light illumination. The change in the capacitance directly alters the potential distribution between the photo-capacitor and the cellular environment. Therefore, the capacitance displacement in the photocapacitor leads to the potential displacement at the interface (ionic charge redistribution across the cell membrane, which leads to cell membrane capacitance displacement) of the neuron with the photocapacitor. If we have an input light pulse U(t) Cos (π/2n), where n is the pulse number and t is the time, the photo-induced dipole rotates between 0 and 90 degrees, and the photocurrent flows in two opposite directions. Like the works that have already been explained before [17,41], we can find the membrane potential related to the input light intensity and polarization [17]:


$$V_{trans}(t)=-Af_{m}/\;A_{jm}\;(Rm\;\cdot I_{device}(t))\;\cdot (\text{1}-e-t/(R_{m}C_{m}))$$ (8)

$$V_{oc}\left(I_{\text{0}},\;\theta \right)=\frac{nkbt}{q}.ln\left(I_{\text{0}}\cdot {cos}^{\text{2}}\left(\theta (t)\right)\right)$$ (9)

$$V_{mpeak}\left(I_{\text{0}},\;\theta \right)=\frac{nkbt}{q}.ln\left(Io\cdot {cos}^{\text{2}}\left(\theta (t)\right)\right)+\beta$$ (10)

We can use V_m, peak_ (I_0_) = −1.14 + 6.5 * ln (I_a_ * cos²(φ)) for PCE12:ITIC after correction based on the α = V_OC_-P3HT/V_OC_-PCE12 = 0.8/0.6 ≈ 1.33. And finally, the time response could be obtained as follows:


$$V_{m}(t)=V_{peak}\cdot e-\frac{t-t_{off}}{\tau }+V_{rest}$$ (11)

$$V_{m}(t)=\alpha \cdot V_{OC}(t)+V_{rest}$$ (12)

#### Dipole-dipole interaction.

Considering that photo-induced dipoles in photocapacitive or nanostructured materials can generate local electric fields [5,42], these fields can induce corresponding dipoles in the nearby cell membrane, which itself behaves as a biological capacitor. This interaction can lead to electrostatic modulation of the membrane potential through the redistribution of ionic charges across the membrane, thereby influencing the cell’s excitability and potentially triggering bioelectrical responses. One example of cellular stimulation through electrostatic force is the use of polyamide 66 embedded with graphene nanoplates for electrostatic polarization of glioblastoma cells [14]. The interaction between photo-induced dipoles (V_dd_) in nanostructured materials and the cell membrane can be described through the electrostatic potential generated by a time-varying dipole moment P(t). The resulting potential at a distance r from the dipole is given by:


$$V_{dd}(t)=\frac{\text{1}}{\text{4}\pi \in_{\text{0}}}\cdot \frac{P(t)}{r^{\text{2}}}$$ (13)

Where ε_0_ is the vacuum permittivity. This potential induces an electric field at the cell interface leading to polarization of the lipid bilayer, and effectively alters the transmembrane potential through charge redistribution. In addition to membrane potential modulation via local dipole fields, the membrane capacitance itself may evolve over time due to charge accumulation and electrostatic deformation of the bilayer. This dynamic behavior can be modeled as [42]:


$$\Delta C_{m}(t)=C_{m}^{\text{0}}(\text{1}+\gamma \frac{Q(t)}{Qref})$$ (14)

Where C_m_ is the baseline membrane capacitance, Q(t) is the time-dependent local charge density, Q_ref_ is a reference charge corresponding to a physiologically relevant scale (e.g., depolarization threshold), and γ is a dimensionless scaling factor reflecting the membrane’s electro-mechanical sensitivity. At nanometer-scale separations, the interaction between a photo-induced dipole in a nanostructured biointerface and an induced dipole on the cell membrane can be described by the dipole–dipole interaction force (F_dd_), governed by:


$$F_{dd}(t)=\frac{\text{1}}{\text{4}\pi \varepsilon_{\text{0}}}.\frac{P\text{1}.P\text{2}.\text{3}(P_{\text{1}}.\hat{r}+P\text{2}.\hat{r})}{r^{\text{3}}}$$ (15)

This force captures both orientation-dependent attraction/repulsion and the dynamic coupling between the dipolar polarization fields. In the context of cellular modulation, this interaction may lead to localized mechanical deformation, redistribution of membrane charges, or even nanoscopic strain fields that influence ion channel gating. The modulation of membrane potential by external fields is a fundamental mechanism in photoelectrical and piezoelectric stimulation.

The change in transmembrane voltage ΔV_m_(t) can be approximated as directly proportional to the induced electric field and the local membrane thickness or displacement:


$$\Delta V_{m}(t)\propto E_{induced}(t)\cdot d_{m}$$ (16)

This relationship reflects how external fields generated by photo-induced surface dipoles, piezoelectric strain, or charge redistribution can penetrate the capacitive membrane structure, altering its potential difference. In soft bioelectronic interfaces, especially those using thin-film semiconductors or quantum dots, spatial field gradients and nanometer-scale dm can amplify this effect, enabling efficient modulation of excitable cells at low stimulation thresholds.

The electrical field induced through the dipole-dipole interaction causes current displacement, and we can find this displacement as follows:


$$I_{disp}(t)=\frac{d}{dt}(P_{\text{1}}(t)\cdot A_{interface})$$ (17)

This formulation highlights the fact that dynamic changes in material polarization, even in the absence of net charge injection, can generate sufficient electrostatic influence to alter the membrane potential of nearby excitable cells.

In capacitive stimulation mechanisms, such as those involving photoactive or piezoelectric biointerfaces, the membrane potential is not altered by charge injection but by displacement currents that charge the cell membrane capacitively. The transmembrane voltage V_m_(t) is governed by:


$$V_{m}(t)=\frac{\text{1}}{C_{m}}\int I_{disp}(t)dt$$ (18)

Here, I_disp_(t) originates from the time derivative of polarization in the adjacent material, and C_m_ is the specific capacitance of the lipid bilayer. This integral relationship shows that the accumulated displacement current, even in the absence of faradaic reactions, can drive significant depolarization of excitable cells, particularly when high-frequency or pulsed stimuli are used.

A more comprehensive description of capacitive stimulation considers both polarization dynamics in the biointerface and ionic currents across the cell membrane. The evolution of the transmembrane potential can be described by:


$$V_{m}(t)=\int_{\text{0}}^{t}\frac{\text{1}}{C_{m}(t)}(\frac{dP(t^{\prime })}{dt^{\prime }}-J_{ion}(t^{\prime }))]dt^{\prime }$$ (19)

Here, dP(t′) represents the time derivative of the polarization vector in the adjacent nanostructured or piezoelectric material, contributing a non-Faradaic displacement current, while J_ion_(t′) accounts for ionic leakage or active transport, such as potassium or calcium channel-mediated currents.

This formulation models the membrane potential V_m_(t) under complex bioelectronic stimulation, where both the polarization dynamics and membrane capacitance evolve over time in response to the accumulated surface charge:


$$V_{m}(t)=\int_{\text{0}}^{t}[\frac{\text{1}}{C_{m}(t)\times (\text{1}+\gamma Q(t^{\prime })/Q_{ref})}\cdot (\frac{d}{dt^{\prime }}(Q(t^{\prime })\cdot d\cdot cos\theta (t^{\prime }))-J_{ion}(t^{\prime }))]dt^{\prime }$$ (20)

The term Q(t′)⋅d⋅cosθ(t′) models the dipole moment aligned between the stimulation interface and the membrane, with θ(t′) denoting the instantaneous angle between the dipole and membrane normal. The denominator models a nonlinear, charge-dependent membrane capacitance, where Cm(t′) increases with surface charge via the scaling term $\left(\text{1}+\gamma \;\frac{Q(t^{\prime })}{Qref}\right)$, reflecting electromechanical modulation of the membrane. J_ion_ (t′) captures ionic leakage or channel currents, acting in opposition to the displacement-induced membrane charging.

Together, this model allows for simulation of light- or force-driven stimulation incorporating field orientation, electrostatic coupling, membrane softening, and active ionic feedback, enabling prediction of neuronal or retinal responses under dynamic stimulation conditions.

#### Dipole-ion interaction mediated by photofaradaic current.

In the semiconducting NPs-polymeric hybrid system, photo-induced local electrical charges can accumulate in a conductive polymer [43], thereby altering the cell membrane potential [42]. One example of this technology is the hybrid of semiconducting NPs and carbon nanotubes [6]. Additionally, the cell membrane can be altered through Faradaic electrochemical reactions and the accumulation of superoxides [44,45]. Photofaradaic charge transfer initiates local ion redistribution by leveraging Fermi-level alignment and surpassing the ionization thresholds of medium-sized species, such as Cl⁻ and water. These interactions modulate the ionic double layer, leading to capacitive displacement or electrochemical gating of the cell membrane potential. The ionization energies and redox potentials of biologically relevant ions, along with their implications for photofaradaic interactions at the bio interface, are summarized in Table 1. The Faradaic current density J_F_(t) describes the charge transfer across the biointerface when electron exchange with the electrolyte or cellular environment becomes energetically favorable. It follows a nonlinear exponential dependence on the interfacial voltage V_s_(t), as described by:

**Table 1:** Ionization energies and redox potentials of biologically relevant ions in aqueous media, highlighting their role in photo-faradaic interactions at the biointerface. These parameters are critical for understanding how photoelectrode Fermi levels facilitate or restrict ion redistribution, membrane depolarization, and capacitive or electrochemical neural stimulation.

Ion/Species	Ionization Energy (eV)	Redox Potential vs SHE (V)	Electrochemical Implication
Na⁺ → Na	5.14	–2.71	Very hard to reduce in water
K⁺ → K	4.34	–2.93	Strong reducing potential
Cl⁻ → Cl₂	~3.6	+1.36	Oxidation possible
H₂O → OH ⁻ /H⁺	~6.5	1.23 (OER)/ 0 (HER)	Major contributor to ionic flux


$$J_{F}(t)=J_{\text{0}}(e^{\beta qVs(t)/kBT}-\text{1})$$ (21)

This formulation accounts for redox reactions at the semiconductor–electrolyte interface and becomes dominant when the interface potential exceeds the threshold for electron injection into electrochemical states (e.g., reduction of ions, ROS generation).

To describe photofaradaic electron transfer at the interface between nanostructured semiconductors and biological media, the Faradaic current density can be expressed in terms of energy level alignment between the material’s quasi-Fermi level E_F,n_ and the redox potential E_redox_ the electrolyte [46]:


$$J_{F}=J_{\text{0}}[e^{\beta (EF,n-E_{redox})}-e^{-(\text{1}-\beta )kTq(EF,n-E_{redox})}]$$ (22)

This formulation captures the asymmetric kinetics of forward (reductive) and reverse (oxidative) electron transfer, with the net current driven by the energy difference between E_F,nE_ and E_redox_.

The modulation of the membrane potential ΔVm under photoelectrical stimulation arises from both capacitive and ionic effects. The first term, Q_photo_/C_m_, represents the capacitive charging of the membrane driven by photo-induced charge accumulation at the interface. This effect dominates in purely capacitive systems where no charge crosses the membrane:


$$\Delta V_{m}\propto \frac{Q_{photo}}{C_{m}}+\sum_{i}\frac{z_{i}e\cdot \Delta [i]}{\varepsilon_{r}}$$ (23)

The second term accounts for ion concentration gradients near the membrane surface, which can emerge due to Faradaic reactions, local electrostatic fields, or ionic redistribution induced by the stimulation. The contribution from each ion species is weighted by its valency z_i_ and scaled by the local dielectric.

#### Dipole-ion interaction mediated by photocapacitive current (indicating the electrostatic coupling between an induced dipole and nearby free or bound charges, e.g., on the membrane surface).

Considering that the photo-induced dipoles in a photo-capacitor interact with the extracellular ionic charges (attracting or repelling extracellular ions), and they rearrange the extracellular ionic charges, which alters the membrane capacitance [47]. We can find dipole interactions with ions (e.g., K ⁺ , Na ⁺ , Ca^2^⁺) as follows:


$$\overset{\to }{F_{ion}}=\nabla (p.\overset{\to }{E})=\frac{q_{ion}}{\text{4}\pi \varepsilon_{\text{0}}\varepsilon_{r}}[\frac{\text{3}(p.\hat{r}-\overset{\to }{p}}{r^{\text{4}}}]$$ (24)

This force redistributes ions and induces capacitive currents and cellular stimulation:


$$I_{dis}(t)=C_{m}\cdot \frac{dV_{m}(t)}{dt}$$ (25)

This model is specifically useful when using QDs for cellular stimulation:


$$V_{m}(t)=\frac{\text{1}}{\text{4}\pi \varepsilon \text{0}\varepsilon r}\frac{\text{2}q\cdot d(t)}{r^{\text{3}}}\cdot \delta (along\;z-axis)$$ (26)

q ⋅ d(t) is the time-dependent photo-induced dipole moment, r is the distance from the QD cluster to the membrane, and δ is the membrane thickness (5 nm).

#### Transmembrane potential modulation with different photocapacitors.

There are various studies on different photocapacitors embedded with different photovoltaic junctions for controlling neuronal transmembrane potential. To compare different studies from both solar cells and photoelectrodes for neural stimulation, different biointerfaces with various open-voltage circuits and the calculated magnitude of the photo-induced dipoles have been listed with the recorded transmembrane potential experimentally from neurons for different studies (see Tables 2 and 3). In some photovoltaic substrates, plasmonic structures are used to enhance the photo-voltage response to different light polarizations.

**Table 2:** The list of different photo-capacitors and calculated photo-induced dipoles for polarization modulation. (See the methodology section supplementary information).

Material	Dipole (a.u.)	Vm @5 Hz (mV)	Vm @50 Hz (mV)	Capacitance Displacement@5 Hz (pC/cm²)	Capacitance Displacement@50 Hz (pC/cm²)	Biocompatibility Score (1–10)	Light Intensity Required (mW/cm²)	Ref
Halide Perovskite (bulk)	8.0	20	4.0	80	20	2	5	[14]
Perovskite QDs (e.g., CsPbBr3)	4.5	12	2.5	55	12	4	10	[48]
ITO/P3HT:PbS QD:PCBM	5.0	15	3.0	60	15	3	8	[17]
CdSe/ZnS QD (core-shell)	4.8	14	2.8	58	14	5	8	[49]
InP/ZnO QD (core-shell)	4.0	11	2.2	51	11	8	6	[28]
PbS/shell QD (core-shell)	6.4	17	3.5	66	17	6	5	[24]
Small Molecules	3.5	9	1.8	40	9	9	12	[50]

**Table 3:** The list of different photovoltaic substrates and calculated photo-induced dipoles for polarization modulation.

Photoelectrodes	Photovoltage (mV)	Distance (nm)	Calculated dipole magnitude (q.d) (pC/cm)	Transmembrane potential	Ref
ITO/Cds/Cds-ZnSe-Cds	600 (100mW/cm^2^)	8.0	100	--------------	[16]
ITO/ZnO/P3HT: Pbs: PCBM (Photocapacitor)	320 (100mW/cm^2^)	49.5	--------------	5mV/1mW *10ms	[11]
ITO/TiO2/InP QDs	150 (50mW/cm^2^)	5.0	--------------	24mV/200mW *10ms	[2]
ITO/ZnO/P3HT: Pbs: PCBM (Pseudocapacitor)	400 (100mW/cm^2^)	49.5	12	41mV/150mW *10ms	[17]

##### Experimental validation of light polarization effects in PCE12:ITIC photoelectrode:

**Fig 3:**
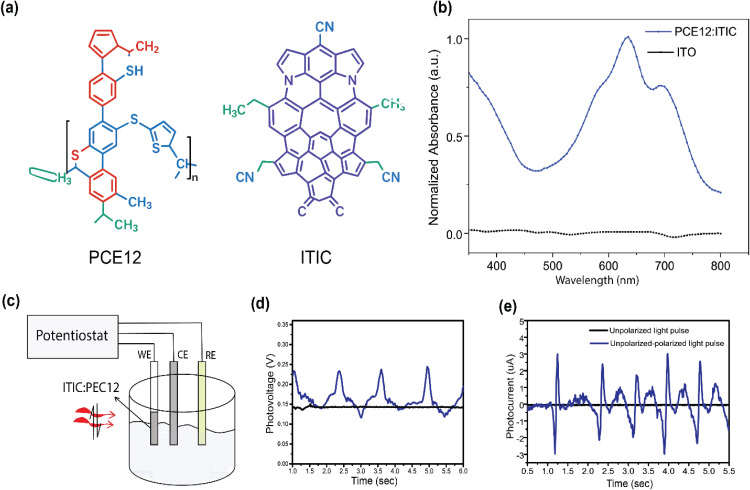
(a) Chemical structure of PEC12:ITIC, (b) Light absorption spectra compared with control. (c) Potentiostat for photocurrent and photovoltage measurements. (d), and (e) Photocurrent response of a PCE12:ITIC polymer blend under continuous polarized light illumination. The device exhibits polarization-dependent enhancement in both photovoltage and photocurrent, indicating that anisotropic molecular orientation and dipole alignment modulate charge separation efficiency. The results highlight the role of excitonic field alignment and photocarrier dynamics in polarization-sensitive organic photovoltaic systems.

The comparison between ITO/ZnO/P3HT:PCBM and ITO/ZnO/PCE12: ITIC device architectures highlights the influence of material selection on open-circuit voltage (V_OC_). While P3HT:PCBM devices typically achieve V_OC_ values of around 0.60–0.65 V, they are limited by strong exciton binding energy and recombination losses due to suboptimal donor–acceptor interfaces. In contrast, PCE12:ITIC blends demonstrate significantly higher V_OC_ values (~0.75–0.90 V), attributed to improved donor–acceptor phase separation, reduced exciton binding energy, and favorable energy level alignment, particularly due to the deeper LUMO level of ITIC. The chemical structure of PCE12 and ITIC is shown in Fig 3a. UV-Vis absorption spectra of the PCE12:ITIC bulk heterojunction thin film are measured using an Agilent Cary 60 UV-Vis spectrometer and compared with those of the Control (ITO) substrate (Fig 3b). The sample exhibits an appropriate absorption peak for analysis with the red laser of wavelength 635 nm.

These features support more efficient charge separation and collection, making PCE12:ITIC blends well-suited for applications requiring enhanced photoresponse and polarization-sensitive stimulation. Fig 3c depicts the potentiostat setup used to record chronoamperometry measurements from our photoelectrodes. The results illustrate the polarization-dependent enhancement in open-circuit photovoltage (V_oc_) and photocurrent for a PCE12:ITIC polymer blend under continuous polarized light, highlighting the role of anisotropic molecular orientation in modulating charge separation efficiency (Fig 3d and 3e). To account for near-zero mean currents, in photocurrent measurements, variability was expressed as a normalized relative standard deviation (RSD%), calculated as the ratio of the standard deviation to the mean absolute current (|I|), providing a more robust measure of signal fluctuation for four different samples of ITO/PEC12:ITIC (Supplementary Figure S2 in S3 File).

**Fig 4:**
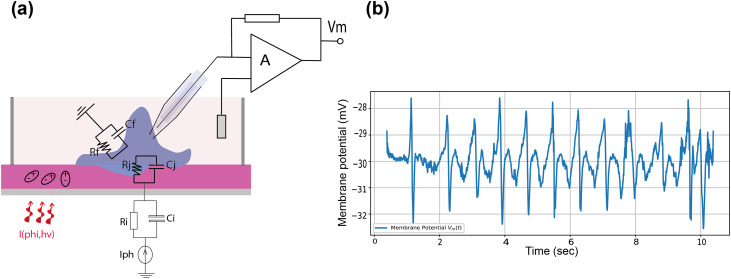
(a) Schematic of whole cell patch clamp measurement of an isolated neuron with electrical circuits, (b) Simulated membrane potential V_m_(t) in response to a time-varying photovoltage V_OC_ (t) derived from a polarized-light-driven PEC12:ITIC photovoltaic device.

As shown in Fig 4, the simulated membrane potential V_m_(t) of an SHSY-5Y cell follows an RC-like exponential response to the time-varying open-circuit voltage V_OC_ generated by a polarized-light-driven PEC12:ITIC photovoltaic interface, illustrating how photovoltaic dynamics modulate membrane charging and bioelectrical stimulation in retinal applications. The model employs an RC-like exponential response to simulate the gradual charging of the SHSY-5Y cell membrane, characterized by a resting membrane potential of −30 mV, thereby highlighting the influence of photovoltaic dynamics on bioelectrical stimulation in retinal interfaces.

##### A comparison study between charge and excitonic stimulation in PCE12:ITIC hybrid:

We modeled the photo-biointerface as a two-stage linear driver feeding a passive membrane. For the **charge model**, light pulses produce an interfacial current density J_in_(t) = J_max_.u(t) (0 ↔ 1 square pulse with 0.2 ms cosine edges), filtered by semiconductor dynamics J_eff_ = (J_in_−J_eff_)/ τ_sc_ with τ_sc_ = 0.5ms and J_max _= 10 A m^−2^. The interface potential obeys C_int_ϕ_0_ + ϕ_0_/R_leak _= J_eff_ with C_int_ = 0.30 F m^−2^C and R_leak _= 0.02 Ω m^2^ (time constant ∼6.7ms). For the **excitonic model**, polarization modulation toggles the incident linear polarization 0° ↔ 90°; exciton-domain normal polarization follows Pn_target _= P_0_Scos (2(θ_pol_ − θ_dom_)) with P_0 _= 0.01 C m^−2^, order S=0.8, θ_dom_ = 0∘, and first-order kinetics Pn=(Pn_target_−P_n_) τ_ex_ = 0.2 ms; the interfacial potential is ϕ_0_ = Pn. In both models the extracellular potential at the membrane includes **Debye screening** and near-field patterning: ϕ_e_(t) = ϕ_0_(t) γ = exp(−d/λD)cos(kx_0_) with λD = 0.8nm, d = 1.2d = 1.2 nm (so exp (−d/λD)≈0.223) and cos (kx_0_) =1 (antinodal placement). The membrane is isopotential and passive: V˙_m_ =−(V_m_ − E_L_)/τm − α_m_ ϕ˙ e with E_L_=−70 mV, τ_m_ = 10ms, α_m_ = 1. We simulated at 100 kHz (forward-Euler), initial conditions ϕ_0_ = Pn = J_eff_ = 0, V_m_ = E_L_. Plotted **five pulses** for each frequency: **1 Hz** (20 ms ON/80 ms OFF over 0.5 s) and **100 Hz** (2 ms/8 ms over 50 ms). As shown in Figure S3 in S3 File, both charge injection and excitonic polarization produce distinct input waveforms and interface potentials at 1 Hz and 100 Hz, highlighting how carrier dynamics and domain polarization shape the extracellular drive reaching the membrane.

The composite Figure S4 in S3 File shows the simulated membrane potential displacement (ΔV_m_) for five light-modulated pulses at 1 Hz and 100 Hz, comparing a charge-injection model (top row) with an **excitonic polarization model** (bottom row). In the charge case, carrier injection into the electrolyte double layer produces large, sharp transients (≈ 20 mV at 1 Hz) that decay fully between pulses. At 100 Hz, the responses partially overlap, yielding smaller ripple-like displacements (~10 mV). In contrast, the excitonic model, where polarization modulation of microdomains drives bound surface charge, produces more symmetric, Debye-screened responses (≈11 mV), with the 1 Hz traces showing well-separated biphasic spikes and the 100 Hz traces showing compressed but still distinct oscillations. The Debye screening factor (exp(–d/λ_D_) ≈ 0.22 for d = 1.2 nm, λ_D_ = 0.8 nm) strongly attenuates both mechanisms, so the observed ΔVm reflects only the near-surface potential that penetrates to the attached cell membrane.

**Fig 5:**
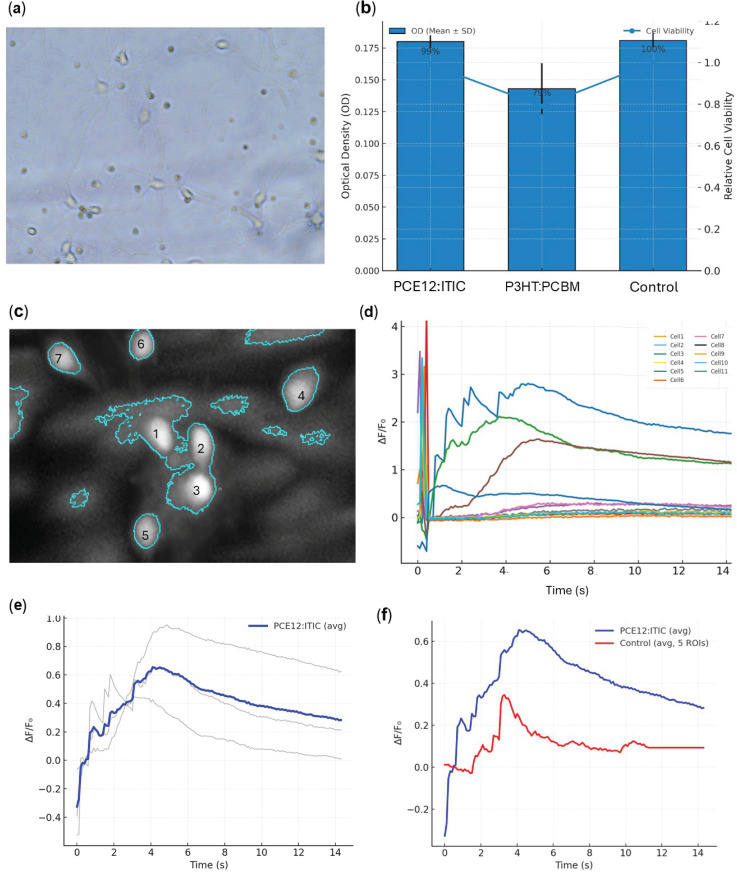
Hippocampal cell culture, viability, and calcium dynamics on organic solar cell substrates. (a) Representative fluorescence micrograph of hippocampal neurons cultured directly on the PCE12: ITIC organic solar cell substrate, showing healthy morphology and network formation. (b) Optical density (OD) measurements across PCE12:ITIC, P3HT:PCBM, and Control groups (bars: mean ± SD), with overlaid relative cell viability normalized to Control. (c) Maximum intensity projection from calcium imaging, highlighting manually selected cell regions of interest (ROIs) with distinct color-coded circles (N = 11). (d) Corresponding ΔF/F₀ calcium transients for the ROIs, demonstrating stimulus-evoked intracellular calcium responses and cell-to-cell variability (N = 11). (e) Calcium fluorescence dynamics recorded from hippocampal neurons cultured on PCE12:ITIC solar cell substrates. Individual cell responses are shown in gray, with the population average highlighted in blue. (f) Average calcium signal difference between a cell grown on top of PCE12:ITIC and on the ITO control substrate.

To assess the compatibility of hippocampal neurons with the organic solar cell substrate, we combined morphological, viability, and functional analyses (Fig 5). Fluorescence microscopy confirmed that cells adhered to and lived on the substrate (Fig 5a), while OD-based viability assays revealed no significant difference between control and substrate conditions, with cell viability maintained above 79% (Fig 5b). Calcium imaging of hippocampal neurons cultured on PCE12:ITIC substrates revealed robust intracellular calcium dynamics in response to optical stimulation. Fig 5c shows the hippocampal cells grown on the PCE12:ITIC, and Figure D shows single cell ΔF/F₀ traces from the PCE12:ITIC sample. The recordings, sampled at 100 ms per frame over a 15 s window, confirm that PCE12:ITIC films effectively mediate optically induced calcium transients at the single-cell level. Individual traces capture cell-to-cell variability, while the population average highlights a consistent increase in intracellular calcium during light stimulation (Fig 5(e)). The recordings demonstrate that PCE12:ITIC films support light-driven modulation of neuronal calcium signaling beyond baseline fluctuations (Fig 5f). While the transparent ITO control occasionally showed artifactual calcium fluctuations due to direct light leakage to the detector, the PCE12:ITIC substrate produced markedly higher and stimulus-locked calcium activity, confirming its photostimulation efficiency.

### The mechanism of excitonic stimulation by photo-induced dipoles in a nanoantenna

#### Harmonic generation in quantum dots and P3HT/PCBM junctions under polarized light modulation.

Organic junctions are typically non-crystalline, resulting in spatially non-uniform photocurrents and photopotentials across the surface. In contrast, nanocrystals possess a crystalline structure, allowing the generation of a more homogeneous electric field under light excitation. It has been demonstrated that the photo-induced dipole emission patterns in core-shell quantum dots (QDs) can be manipulated by varying the incident light angle, and this effect can also be achieved through polarization modulation of the light. A comparison of photo-induced dipole magnitudes in semiconductor nanocrystals, metallic nanoparticles, and carbon-based particles, measured via Kelvin probe techniques, is provided in Table 4. The Hamiltonian of an electron-photon interaction could be written as follows:

**Table 4:** The comparison of the photo-induced dipoles in semiconductor NCs and in metallic and carbon particles measured with the Kelvin probe.

Photo-electrodes	Radius	Photovoltage	Ref
InN/InGaN	11nm	150mV	[51]
Gold NPs	20nm	14 mV	[52]


$$\text{H}(\text{t})=\text{H}+{\text{e}}_{\text{r}}.\;\vec{\text{E}}(\text{t})$$ (27)

For linear light polarization, the electric field could be obtained as follows:


$$E(t)=\varepsilon_{\text{0}}\left[\text{0},\text{0},Cos(\omega t)\right]$$ (28)

For circular light polarization, the electric field could be obtained as follows:


$$E(t)=\varepsilon_{\text{0}}\left[\text{0},\text{0},Cos(\omega t),\;Sin(\omega t),\text{0}\right]$$ (29)

In fact, with the light polarization modulation, we can manipulate the electron-hole wave function of a nanocrystal. Equations 32 and 33 show the sorundinger equations for two linear and circular light polarizations:


$$\{\;-\frac{h^{\text{2}}}{\text{2}}\frac{\partial }{\partial t}\frac{\text{1}}{mz(z)}\frac{\partial }{\partial z}+V(z,t)\}\varphi_{n}(z)=E_{n}\varphi_{n}(z,t)+\;e.\varepsilon_{\text{0}}.z.cos(\omega t)$$ (30)

$$\{\;-\frac{{\text{h}}^{\text{2}}}{\text{2}}\frac{\partial }{\partial \text{t}}\left(\frac{\text{1}}{\text{mx}(\text{x})}\frac{\partial }{\partial \text{x}}+\frac{\text{1}}{\text{my}(\text{y})}\frac{\partial }{\partial \text{y}}\right).\text{V}(\text{x},\text{y},\text{t})\}\varphi_{\text{n}}(\text{x},\text{y})={\text{E}}_{\text{n}}\varphi_{\text{n}}(\text{x},\text{y})+\frac{\text{1}}{\text{2}}\;\left(\text{e}.\epsilon_{\text{0}}.\text{x}.\text{Sin}(\omega \text{t})+\text{e}.\epsilon_{\text{0}}.\text{y}.\text{cos}(\omega \text{t})\right)$$ (31)

In metal particles such as gold particles, the photovoltages have been shown to be varied by the light polarization and also the electrochemical current (photocatalysis activity) has been shown to be enhanced by the increase of the light polarization angle (it is maximum when the light is prependicular to the particle surface) [18].

To find the effective capacitance displacement due to the photoinduced dipoles in QDs to the cell membrane, we can use the following equation:


$$\frac{dV_{m}}{dt}=\frac{NQD\cdot A\cdot sin(\text{2}\pi ft)-\frac{V_{m}}{R_{m}}}{C_{m}}$$ (32)

#### Electric dipole oscillator through light polarization modulation in bulk photocapacitors.

With the polarization modulation of light, an oscillating photo-induced dipole induces a radiation of electric field (gradient of electric field) at the surface of an InP/ Zinc Sulfide (ZnS) Quantum dot or the ITO/Zinc oxide (ZnO)/P3HT: PCBM bio-interface. Equation 35 describes an oscillating photo**-**induced dipole, while I_a_ represents the oscillating charges that cause the radiation of an electric field. This facilitates the generation of an extracellular oscillating electric field through a capacitive bio**-**interface or a core**-**shell quantum dot. The potential because of the dipole radiation can be represented as follows:


$$V_{d}(r,t)=\frac{P_{\text{0}}Cos\theta }{\text{2}\varepsilon_{\text{0}}\lambda r}Sin\left(\frac{\text{2}\pi c\left(t-\frac{r}{c}\right)}{\lambda }\right)$$ (33)

$$\frac{dV_{m}(t)}{dt}=\frac{A\cdot sin(\text{2}\pi ft)-\frac{V_{m}(t)}{R_{m}}}{C_{m}}$$ (34)

Where I_a_ is the amplitude of photo-induced charges’ displacements, ${\text{P}}_{\text{0}}=\text{q}\times \text{d}$ is the amplitude of the photo-induced dipole, and r is the distance from any node to the point source.

**Fig 6:**
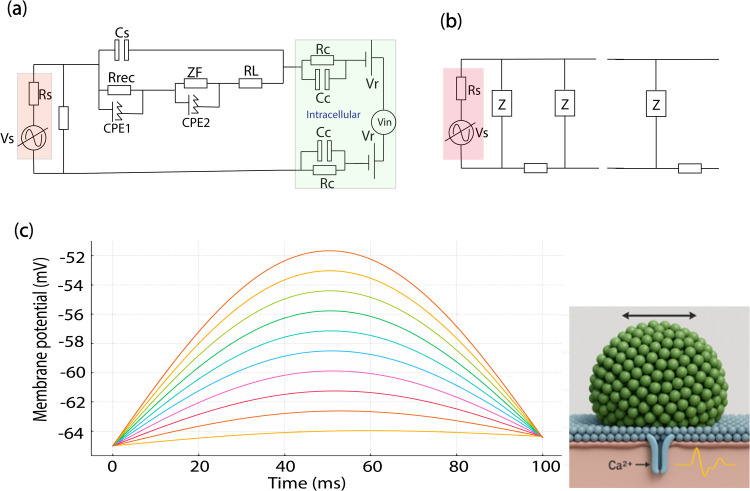
Polarization modulation for the photo-stimulation of cells with photo-induced electrical dipoles in a photo-capacitive substrate, (a) electrical circuit model for the photoelectrical stimulation of cells through a photocapacitor, (b) Transmission line model for neural stimulation through the radiation of a photo-induced dipole with polarized light effect, (c) Membrane potential induced by a cluster of QDs (nano antennas changing from 5-100 numbers) in contact with a passive cell membrane with the frequency excitation of 5 Hz. As it is clear, the electric field distribution depends on the frequency of the polarization modulation.

As illustrated in Fig 6, increasing the frequency of polarization modulation leads to more localized and intensified electric field gradients across the membrane surface, affecting the induced transmembrane potential.

### Spatially patterned stimulation (pixel-wise control)

**Fig 7:**
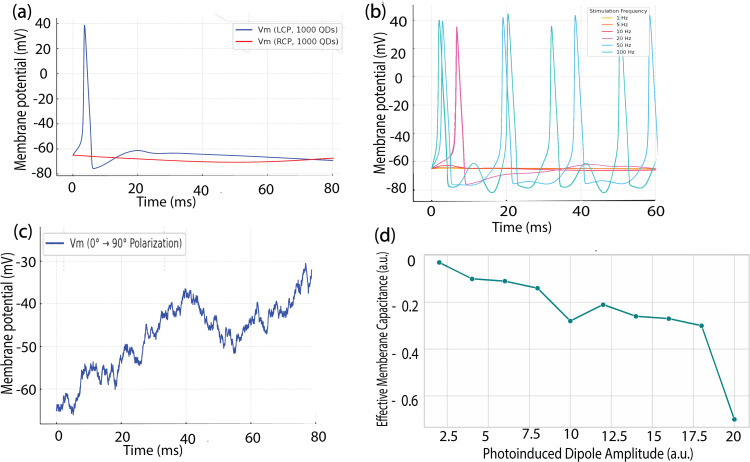
Photoinduced dipole modulation of membrane potential and effective capacitance based on polarization modulation of the light. (a) Simulated membrane potential dynamics under left- and right-circularly polarized light (LCP vs. RCP), demonstrating asymmetric stimulation due to dipole orientation. (b) Hodgkin-Huxley membrane potential response under dipole-induced capacitive stimulation across multiple frequencies (1–100 Hz), highlighting frequency-dependent spiking behavior. (c) Gradual membrane depolarization under continuously rotating polarization (**0° → 90°**), showing cumulative excitatory effects from spatiotemporal dipole alignment. (d) Extracted effective membrane capacitance as a function of photoinduced dipole amplitude, revealing nonlinear charge displacement and field-membrane coupling.

We also investigated the response of the QD clusters to the left- and right-circularly-polarized (LCP/RCP) for future potential of visual signal processing and selective stimulations based on polarization modulation. Fig 7(a) shows the response of the QD clusters to input LCP and RCP light polarization modulation. In this way, LCP-polarized light would activate excitable retinal areas, thereby mimicking the selectivity of photoreceptors. As illustrated in Fig 7(b), increasing the polarization modulation frequency produces more localized and intensified electric field gradients across the membrane surface, thereby the induced transmembrane potential. The potential applications of polarization-modulated quantum dot interfaces in retinal stimulation and broader neuroprosthetic strategies are summarized in Tables 5 and 6, highlighting both cell-type-specific responses and anisotropic modulation patterns for functional encoding. The biophysical and stimulation parameters used in the simulations, including Hodgkin-Huxley conductance and photo-induced dipole inputs, are summarized in Table 7. In a passive RC membrane model subjected to polarization modulation from 0° to 90°, the membrane potential exhibits a characteristic envelope-shaped waveform. During the 0° phases, where the dipole is aligned with the electric field (cosine projection), the stimulation is strongest, leading to maximal displacement currents and membrane charging. As the polarization shifts toward 90° (sine phase), the dipole orientation becomes orthogonal to the membrane surface, reducing the effective field strength and altering the timing of the capacitive response. This angular modulation results in periodic fluctuations in membrane potential, as the cell integrates varying electrostatic inputs over time (as shown in Fig 7(c)). The observed waveform reflects the dynamic coupling between light polarization, photoinduced dipole alignment, and electrostatic field orientation at the bio-interface.

**Table 5:** Potential application of the polarization modulation for retinal application [53].

Strategy	Polarization	QD Type	Cell Target	Response
Focal excitation	LCP	InP/ZnO	ON-RGCs	Spiking
Surround suppression	RCP	InP/ZnO	OFF-Bipolars	Silent
Edge enhancement	Linear	InP/ZnO	Horizontal Cells	Subthreshold Vm
Safe baseline	No polarization	—	All	No activation

**Table 6:** Application table for the anisotropic engineered structure [54].

Modulation Pattern	Neuro-Application
0°/ 90° switching	On/off gating (retina, cortex)
LCP/ RCP encoding	Directional signaling
Frequency + polarization	Visual prosthetics or temporal coding
Spatial polarization gradients	Tactile or sensory encoding

**Table 7:** Parameters used in the simulations [55,56].

Parameter	Value	Description
C_m_	1.0 µF/cm²	Membrane capacitance
R_m_	10 kΩ·cm²	Membrane resistance (passive RC model)
g_Na_	120 mS/cm²	Sodium conductance (HH model)
g_K_	36 mS/cm²	Potassium conductance (HH model)
g_L_	0.3 mS/cm²	Leak conductance (HH model)
E_Na_	50 mV	Sodium reversal potential
E_K_	−77 mV	Potassium reversal potential
E_L_	−54.387 mV	Leak reversal potential
Initial V_m_	−65 mV	Resting membrane potential
Dipole amplitude	20.0 (a.u.)	Maximum simulated dipole-induced current amplitude
Dipole frequency	200 Hz	Base frequency of dipole oscillation
Polarization modulation freq.	10 Hz	Switching frequency for polarization angle
Polarization angles	0°, 45°, 90°	Angles used in cosine/sine field modulation
Simulation duration	100 ms	Total simulation time
Time resolution	10,000 steps	Number of time points for solution evaluation

For a photocapacitor-coupled cell membrane illuminated with 0° and 90° polarized light at adjacent positions, the electric field gradient across the membrane patch is given by:


$$F=-\nabla (\mu_{mem}\cdot E_{photo}(t))$$ (35)

$$C_{m}(t)=C_{m\text{0}}(\text{1}+\chi \cdot \mu_{mem}\cdot E(t))$$ (36)

Effective membrane capacitance C_eff_ can be estimated as a function of the photoinduced dipole amplitude, derived from simulated membrane potential responses. The estimation follows the proportional relationship $C_{e\text{ff}}\propto \frac{\Delta \text{Q}}{\Delta \text{V}}$, which, under photoelectrical stimulation, can be approximated as C_eff _∝ Dipole Amplitude. At low dipole amplitudes, the membrane exhibits minimal potential shifts, resulting in apparently lower capacitance values due to limited charge displacement. As the dipole amplitude increases, the membrane potential response becomes more pronounced, reflecting enhanced interaction between the induced electric field and the lipid bilayer. This leads to a greater accumulation of charge across the membrane, effectively increasing the estimated capacitance. The nonlinear rise in C_eff_ at higher amplitudes indicates that membrane charging is strongly dependent on the strength of the applied dipolar field, highlighting the electrostatic sensitivity of the system under polarized optical excitation.

As shown in Fig 7(d), the amplitude of the photoinduced dipole increases, the resulting electrostatic field at the membrane interface becomes stronger, driving greater displacement of ionic charges in the surrounding electrolyte. This enhanced charge redistribution contributes to an effective increase in membrane capacitance, or more precisely, a greater capacitive displacement. However, this relationship is inherently nonlinear, as the membrane acts as a voltage-dependent filter that modulates the extent of charge displacement. At higher dipole amplitudes, the membrane’s nonlinear dielectric response and ion channel gating effects begin to dominate, introducing saturation or threshold-like behaviors in the capacitive response. In the simulation, we varied the photoinduced dipole amplitude from 2 to 20 arbitrary units to mimic the strength of dipole fields generated by semiconductor materials (e.g., quantum dots or organic solar cells). The effective membrane capacitance was computed as a synthetic, non-monotonic function representing how the lipid bilayer responds electrostatically to increasing dipole strength. This reflects realistic biophysical behavior, where low dipole amplitudes induce weak membrane charging, mid-range amplitudes enhance capacitive coupling, and high amplitudes lead to dielectric saturation or ionic shielding. The simulated capacitance values ranged from −0.03 to –0.70 a.u., consistent with experimental trends in photocapacitive stimulation. The simulations were conducted using experimentally validated biophysical parameters, including membrane capacitance, resistance, and ion channel conductance, as detailed in Table 7.

## Methods

### Preparation of photoactive blend and photoelectrode fabrication

Two photoactive material systems were evaluated to characterize their optoelectronic properties and select the most suitable system for this study. The first combination was poly(3-hexylthiophene-2,5-diyl) (P3HT) and phenyl C61 butyric acid methyl ester (PCBM), a material most studied for the study of photovoltaic devices and photoelectrodes. P3HT:PCBM photoactive blends were prepared by mixing 12.5 mg of P3HT and 12.5 mg of PCBM in 1 mL of o-dichlorobenzene. The second combination consists of poly[(2,6-(4,8-bis(5-(2-ethylhexyl)thiophen-2-yl)-benzo[1,2-b:4,5-b’]dithiophene))-alt-(5,5-(1’,3’-di-2-thienyl-5’,7’-bis(2-ethylhexyl)benzo[1’,2’-c:4’,5’-c’]dithiophene-4,8-dione))] (PCE12) and 3,9-bis(2-methylene-(3-(1,1-dicyanomethylene)-indanone))-5,5,11,11-tetrakis(4-hexylphenyl)-dithieno[2,3-d:2’,3’-d’]-s-indaceno[1,2-b:5,6-b’]dithiophene (ITIC), a high performing photovoltaic material system. PCE12:ITIC photoactive blends were prepared using 10 mg of each in a 1:1 blend ratio with a concentration of 20 mg/mL in o-dichlorobenzene, and an additive named 2-(2-Thienylmethylene)indane-1,3-dione (SA4) was added afterwards 16% by weight. All of these photoactive blends were stirred overnight at 60 °C in a hood under dark conditions before being used for fabrication. All the materials were purchased from Ossila Ltd.

All devices in this study were fabricated in an inverted geometry with the structure ITO/ZnO (70 nm)/photoactive layer (100 nm) [17,57,58]. The protocol for device fabrication is similar to that reported elsewhere. For this, first, precleaned and patterned ITO-coated glass substrates were plasma-treated for 1 min with 70 W power using an air–plasma system. Thereafter, a sol–gel ZnO solution was spin-coated at 2000 rpm for 60 s to obtain a 70 nm ﬁlm [59]. The substrates were then annealed at 160 °C for 15 min to remove excess solvents. Processing conditions for the different photoactive blends are as follows: 1) P3HT:PCBM photoactive blend was spin-cast at 400 rpm for 100 s in ambient and was allowed to dry for 15 min on a hot plate in air [60], 2) PCE12:ITIC photoactive blend was spin-casted at 2000 rpm for 60 s in the air, and the ﬁlms were annealed at 100 °C for10 min [61,62].

### Electrochemical measurements

Electrochemical impedance spectroscopy (EIS) in frequency response analysis (FRA) potential scan mode was performed on an Autolab Potentiostat Galvanostat PGSTAT (Metrohm, Netherlands). A three-electrode setup consisting of Ag/AgCl as the reference electrode, a platinum wire as the counter electrode, and the photoelectrode as the working electrode was used. The experiment was carried out at room temperature in an aCSF aqueous medium medium as the supporting electrolyte solution. A handheld red laser (635 nm) was directed at the photoelectrode through a linear polarizer. The polarization angle was manually switched between 0° (parallel) and 90° (perpendicular) relative to the device axis. The illumination power density was maintained at 5 mW/cm^2^.

### Cell culture

GIBCO Primary Rat Hippocampus Neurons were used for MTT assay and Ca^++^ imaging experiments which are isolated from day-18 rat embryos and cryopreserved in liquid nitrogen. Each vial contains 1 × 10^6^ viable cells⁄mL cells. The photoelectrode and the ITO control were sterilized with deionized water for 2 hours, followed by 70% ethanol, then treated with UV-C for 5 min. The photoelectrodes and control were treated with poly-D-lysine diluted in PBS at a 1:1 ratio for 1 hour, followed by and then rinsing with sterilized deionized water three times. The samples were left uncovered in the culture hood to dry for 2 hours. A 15-mL conical culture tube was rinsed with pre-warmed (37°C) complete Neurobasal Plus Medium and left in the cell culture hood prior to thawing the cells. The frozen vial was rapidly thawed (< 2 minutes) by gently swirling it in a 37°C water bath. The vial was transferred to the cell culture hood and disinfected with 70% isopropanol alcohol. The thawed sample was transferred to the 15 mL tube; and the vial was rinsed with 1 mL of pre-warmed complete Neurobasal™ Plus Medium (37°C) which was then added to the cells in the 15 mL tube extremely slowly, added dropwise (≈1 drop/s). The suspension was mixed by gentle swirling after each addition. Approximately 150 µL of the cell suspension was spread onto the coated sample surface (substrate in the culture dish) and left to stand in the culture hood for 20 minutes. Then, 3 mL of complete Neurobasal Plus Medium was added to the culture dish. The cells were incubated at 37°C in a humidified atmosphere of 5% CO_2_ in air. The cells were fed after 4–24 hours, and subsequently, every third day by aspirating half of the medium from each well and replacing it with fresh medium until the sample was used for calcium imaging after 7 days of cell growth.

### Biocompatibility test

The cytotoxic effect of photoelectrodes on hippocampal neuron cells was assessed by measuring mitochondrial activity with the MTT assay. Briefly, the photoelectrodes and ITO control were sterilized with 70% ethanol and then treated with UV-C for 5 min. Sterilized samples were placed in a six-well cell culture plate. The MTT assay was performed using the Cell Proliferation Kit I (Sigma-Aldrich, Massachusetts, USA) according to the manufacturer’s instructions. Briefly, primary hippocampus neurons (1 × 10^5^ cells per sample) were seeded on the substrate supplemented with Neurobasal medium and incubated for 48 hours in a 6-well plate. After 48 h incubation, the media were replaced with 1 mL of MTT solution (5 mg/mL in PBS, pH 7.4) and 4 mL of Neurobasal™ Plus Medium mixture per well. The cells were incubated at 37 °C and 5% CO2 for an additional 4 h. The medium was then removed from each well, and the substrates were transferred to an empty six-well plate. In each well, a 1:1 mixture of DMSO and ethanol was added to dissolve the formazan crystals, and the solution was transferred to a 96-well plate for absorbance measurement. The absorbance was measured using a SpectraMax iD3 (Molecular Devices, California, USA) at 570 nm, and the relative cell viability was calculated as follows: Viability = (ODsample/ODcontrol) × 100.

### Calcium Imaging

Hippocampal neurons were cultured on conductive substrates (control ITO and experimental PCE12:ITIC photovoltaic films) for calcium imaging experiments. Intracellular calcium dynamics were monitored using a Leica DM IRB epifluorescence microscope equipped with a high-speed digital camera. Cells were loaded with a calcium-sensitive fluorescent dye (Fluo-4 AM, Thermo Fisher Scientific) at 5 μM in culture medium and incubated for 30 min at 37 °C, followed by a 30-min de-esterification period in dye-free medium. Excitation was delivered using a xenon/LED illumination source with appropriate FITC filter sets (excitation 488 nm, emission 510–550 nm).

Time-lapse image sequences were acquired at a frame interval of 100 ms (10 Hz), yielding a total recording window of 15 s (150 frames). For certain analyses, the first six frames (0.6 s) were excluded to minimize motion artifacts prior to light stimulation onset. Fluorescence intensity was quantified from manually or semi-automatically selected regions of interest (ROIs) corresponding to individual neuronal somata. Raw fluorescence traces F(t) were normalized to baseline fluorescence (F0, median of the first 10 frames) to yield relative calcium changes as ΔF/F₀.

Photovoltaic stimulation was applied by polarized light pulses directed onto the cell–substrate interface. Control ITO samples and active PCE12:ITIC solar cell substrates were analyzed under identical conditions. For each recording, single cell ΔF/F₀ traces were extracted and ensemble averages were computed to compare population responses.

### Computational modeling and Python simulation

A custom Python framework was developed to simulate the effects of photo-induced electric dipoles on cellular membrane potential and the distribution of transmembrane electric fields. The model combines time-dependent photocurrent injection, capacitive charging dynamics of the membrane, and electrostatic force calculations and is available in Supplementary Python codes.

## Conclusion

This study presents a unified model of photoelectrical stimulation driven by photo-induced dipoles in organic and quantum dot–based photovoltaic junctions. Through integrated theoretical modeling, circuit simulations, calcium imaging experiments, and comparative analyses, we demonstrate how excitonic and electrostatic processes, modulated by light polarization, govern the dynamics of transmembrane potential and intracellular calcium fluctuations in hippocampal cells was cultured on the PCE12:ITIC. Among the tested materials, non-fullerene acceptors such as PCE12:ITIC demonstrated superior exciton dissociation and near-IR response, directional dipole alignment, and polarization-sensitive photocurrents, which translated into robust, stimulus-locked calcium transients at the single-cell level. Quantum dot clusters further enhanced control, with LCP/RCP light selectively activating specific domains to mimic native photoreceptor coding. These findings establish a framework for engineering high-resolution, biocompatible photo-capacitor interfaces that couple photonic inputs to neuronal excitability, with translational potential in retinal prostheses and minimally invasive neural therapies.

## Supporting information

S1 File Photocurrent plot in the Fig 3. (XLSX)

S2 File PhtocurrentVsIntensity. (XLSX)

S3 File Supplementary information. (DOCX)

S4 File Supplementary_python_codes. (PDF)
