# Supplementary material for: Mechanistic insights into excitonic and electrostatic stimulation of cells by photovoltaic substrates/nanocrystals and through light polarization modulation
Source: PLoS One. 2025 Nov 7;20(11):e0335978. doi: 10.1371/journal.pone.0335978 (PMC12594350; doi:10.1371/journal.pone.0335978)
Supplement: S3 File — (DOCX) [file pone.0335978.s003.docx]

Mechanistic Insights into Excitonic and Electrostatic Stimulation of Cells by Photovoltaic Substrates/Nanocrystals and through light Polarization modulation

Mohammad Mohammadiaria^1*^, Daniel L. Rathbun^2^, Moses Kamita^3^, and [Shashi Bhushan Srivastava](https://journals.aps.org/search/field/author/Shashi%20Bhushan%20Srivastava)^2^*

^1^Independent Researcher, Pavia, Italy

^2^Henry Ford Health + Michigan State University Health Sciences, Detroit, Michigan, USA

^3^Department of Hematology/Oncology, Henry Ford Health, Detroit, Michigan, USA.

*Corresponding author. E-mail: [momoaria990@gmail.com](mailto:momoaria990@gmail.com); [ssrivas4@hfhs.org](mailto:ssrivas4@hfhs.org)


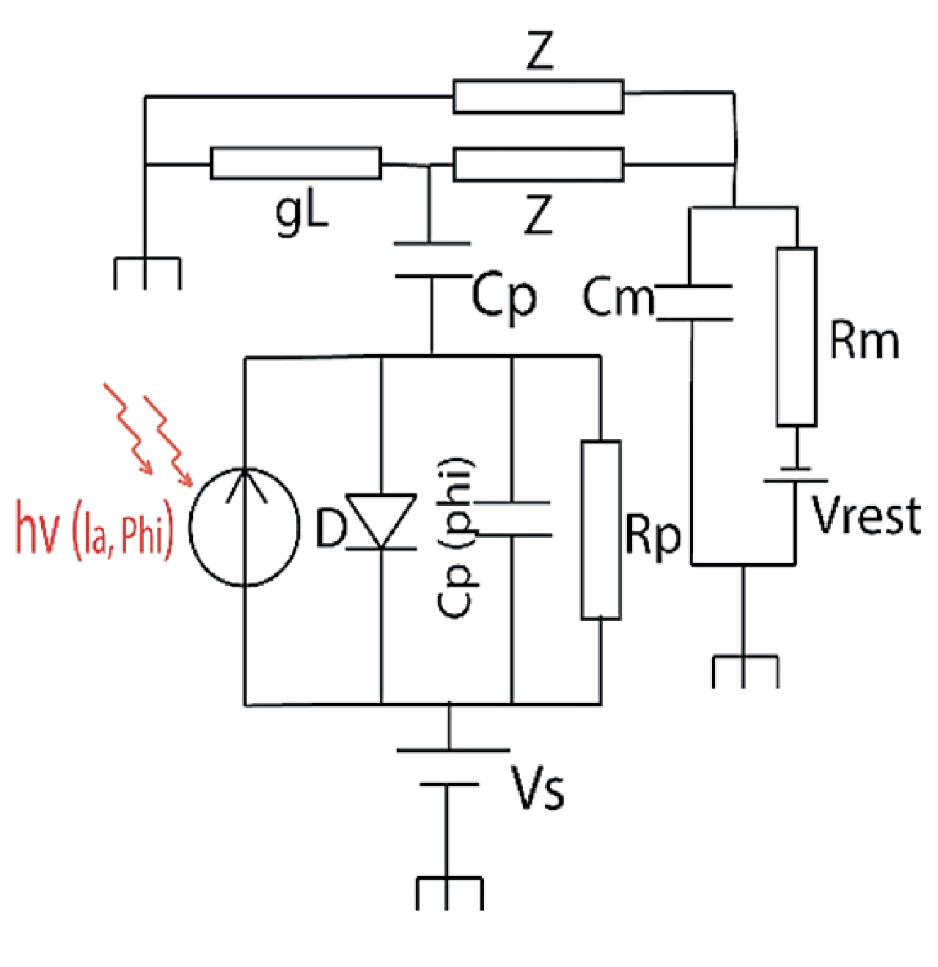
**Supplementary Information**

**Figure S1.** An electrical circuit model of a solar cell, which has been integrated with a passive RC cell model.


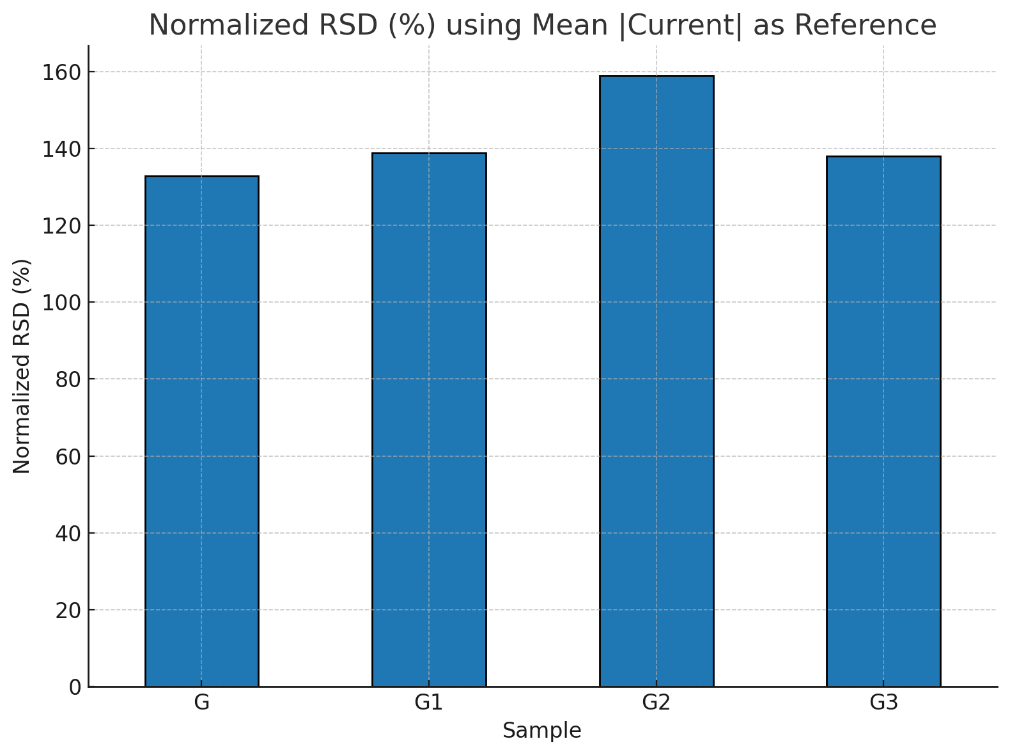


**Figure S2. Normalized variability of photocurrent across samples.**Bar plot of **Normalized RSD (%),** computed as RSDnorm=σI⟨∣I∣⟩×100, for the four datasets (**G**, **G1**, **G2**, **G3**). Using the mean absolute current ⟨∣I∣⟩ as the reference mitigates the near-zero mean bias and provides a robust comparison of signal fluctuations across samples. Higher values indicate larger fractional variability relative to the typical current magnitude.


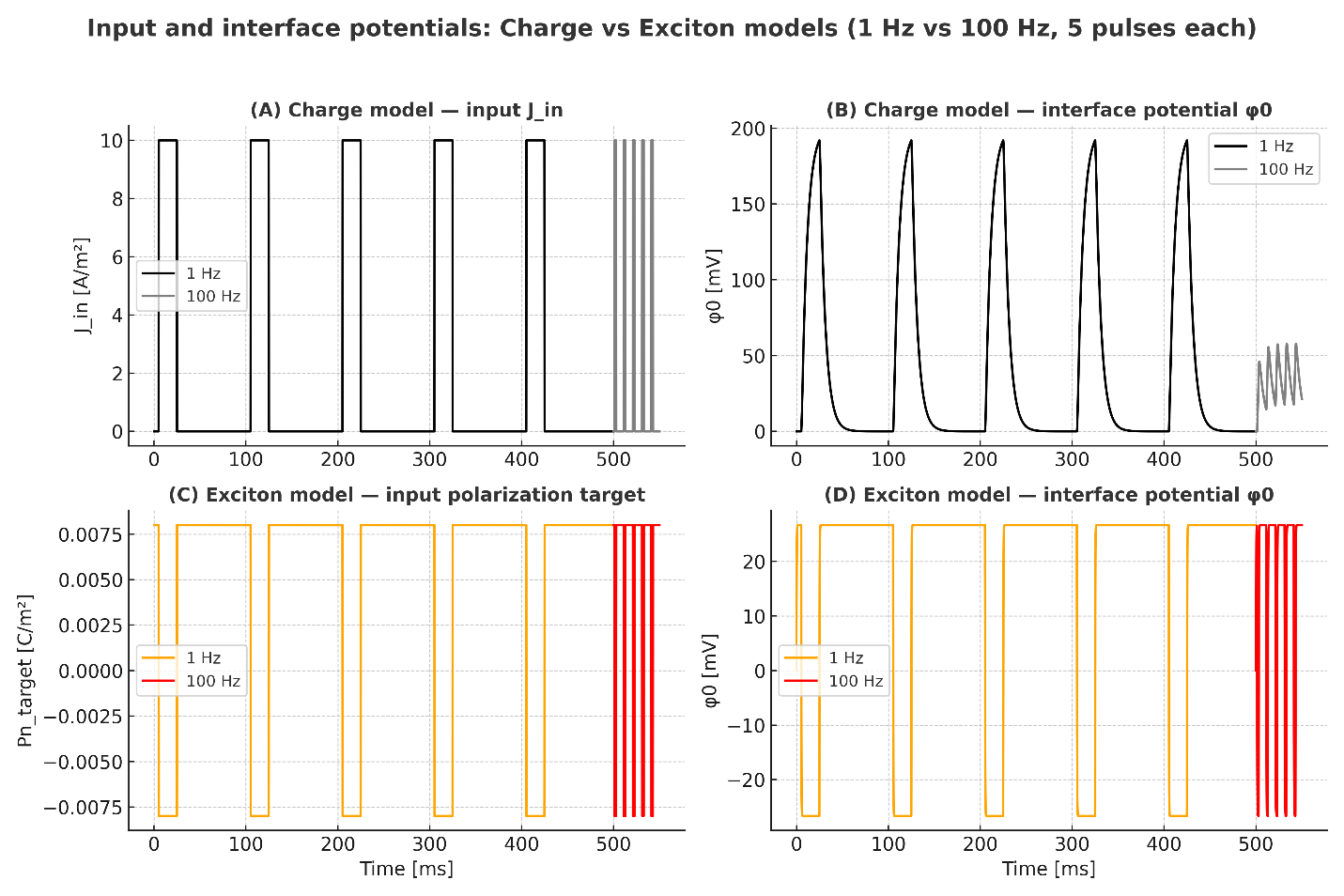


**Figure S3. Input signals and interface potentials for charge- and exciton-driven stimulation.**
Simulated driving inputs and resulting interfacial potentials at the semiconductor–electrolyte boundary for both models under 1 Hz (20 ms ON / 80 ms OFF, black/orange) and 100 Hz (2 ms ON / 8 ms OFF, grey/red) stimulation, each plotted for five pulses. (A) Charge model input: interfacial photocurrent density Jin(t). (B) Charge model interface potential ϕ_0_(t), obtained from capacitive charging of the double layer. (C) Excitonic model input: polarization target Pntarget(t) modulated by incident light polarization. (D) Excitonic model interface potential ϕ_0_(t)=Pn ​/Cint​, reflecting bound surface charge density. In both cases, Debye screening and interface capacitance constrain the effective extracellular potential that subsequently drives the membrane.


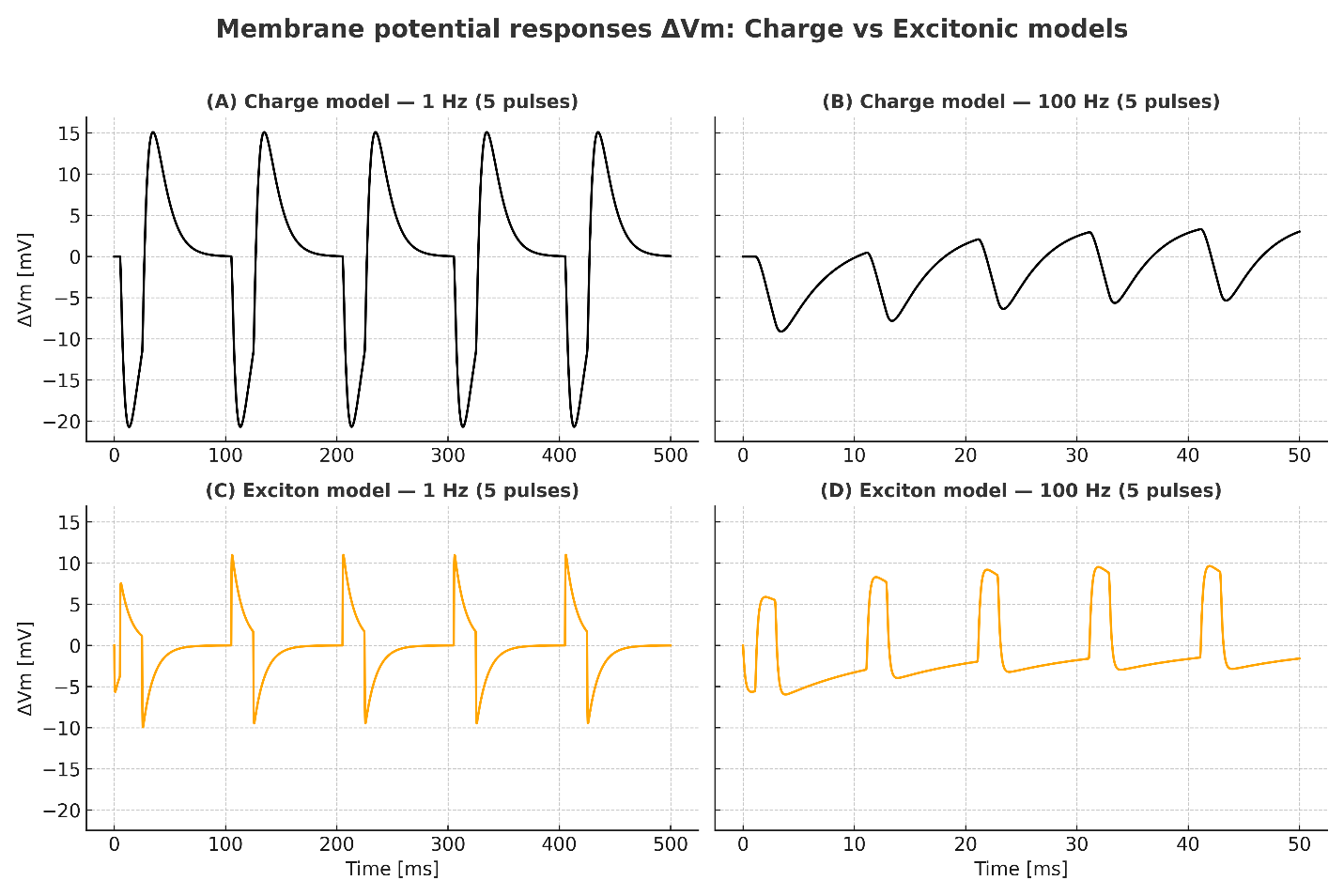


**Figure S4. Membrane potential responses under charge- and exciton-driven stimulation.**
Simulated transmembrane potential displacement (ΔV_m_) for five consecutive pulses at 1 Hz (left) and 100 Hz (right). Top row: **charge-injection model**, where photogenerated carriers accumulate at the semiconductor–electrolyte interface and drive capacitive currents into the membrane. Bottom row: **excitonic polarization model**, where polarization modulation of exciton microdomains induces bound surface charges that couple capacitively through the Debye layer.

**Methodology for Table 2.**

For Table 2, the “Dipole (a.u.)” quantifies a device-relevant, interfacial polarization proxy inferred from the measured/target displacement–voltage pairs. Specifically, we model the photocapacitive biointerface as a single-pole small-signal element, $V_{m}(f) = A /\sqrt{(1 + {(2\pi f \tau)}^{2}} )$ which is the potential difference between photoelectrode and the reference electrode, and estimate τ and A from the two anchor points at 5 Hz and 50 Hz; the corresponding displacement charge density follows ΔD(f) ≈ C_eff_(f) · V_m_(f). Considering a patch of the cell membrane has been attached on the photocapacitor, we can also find the transmembrane potential as ΔV_mem_​(f) ≈ α(f)V_ext_​(f) where α is the transfer factor. To compare materials while limiting frequency-dispersion bias, we form a displacement-per-voltage metric $\phi= \sqrt{(\Delta D5 / Vm,5) (\Delta D50 / Vm,50)]}$ it by the optical drive required to reach the stated V_m_ via a mild intensity factor ${(I_{\mathrm{ref}} /I_{\mathrm{mat}})}^{\gamma}$ with γ = 0.5. And where $I_{ref}=5 mW/{cm}^{-2}$. We then apply a class/orientation factor S_class_ ∈ [0.6, 1.0] to reflect known differences in packing, screening, and ligand/trap landscapes among bulk perovskites, QDs, polymer:QD blends, and small-molecule films. The resulting dipole index is defined as ${DI= \phi.(I_{\mathrm{ref}} /I_{\mathrm{mat}})}^{\gamma}$· S_class_, and the reported Dipole (a.u.) is obtained by a fixed scaling Dipole (a.u.) = K · DI, where K is chosen such that bulk halide perovskite equals 8.0 a.u.; all other entries follow without hand-tuning. This construction preserves the observed ordering of interfacial polarization strength across materials, penalizes higher light budgets for the same bioelectrical response, and yields a comparable, geometry-agnostic index suitable for polarization-modulated neuromodulator design.

ΔD ≈ ΔP ∝ n · μ_eff_ · S · (E_int_ / E_0_)

At small signal, the interfacial displacement charge density (ΔD) tracks the change in interfacial polarization (ΔP). Here, n is the number density of polar motifs, μ_eff_ their effective dipole moment, S an orientation/packing factor, Ei_nt_ the local internal field felt by the dipoles, and E_0_ a reference field scale. Under fixed geometry/electrolyte, ratios across materials allow ΔD/Em to act as a proxy for μ_eff_ · S.
