# Supplementary material for: Mechanistic insights into excitonic and electrostatic stimulation of cells by photovoltaic substrates/nanocrystals and through light polarization modulation
Source: PLoS One. 2025 Nov 7;20(11):e0335978. doi: 10.1371/journal.pone.0335978 (PMC12594350; doi:10.1371/journal.pone.0335978)
Supplement: S4 File — (PDF) [file pone.0335978.s004.pdf]

Supplementary Information: Python Codes

Supplementary Information: Code for Simulated Figures

Author: Dr.Mohammad Mohammadiaria

This supplementary document contains Python code used to generate the simulated graphs presented in the main manuscript. The models include:

Passive RC membrane models under polarization-modulated dipole stimulation (0°–90°, LCP/RCP).

Hodgkin–Huxley (HH) active neuron simulations with input currents derived from light-induced capacitive displacement.

Comparative responses to LCP vs. RCP stimulation, demonstrating threshold crossing and action potential firing.

Customizable parameters for quantum dot dipole strength, membrane coupling efficiency, surface attac

---

```
# Figure6.a
import numpy as np
import matplotlib.pyplot as plt
# --- Simulation parameters ---
fs = 10000           # Sampling rate (Hz)
T = 0.1              # Total time (s)
t = np.linspace(0, T, int(fs * T))
f_mod = 5            # Polarization modulation frequency (Hz)
# --- RC membrane parameters ---
Cm = 1e-6            # Capacitance (F/cm^2)
Rm = 1e7             # Resistance (Ohm·cm^2)
# --- Physical constants ---
epsilon_0 = 8.854e-12  # Vacuum permittivity (F/m)
epsilon_r = 80         # Relative permittivity (water)
r = 4e-9               # QD–membrane distance (m)
lambda_D = 1e-9        # Debye length (m)
screening_factor = np.exp(-r / lambda_D)
# --- Dipole parameters ---
dipole_amplitude = 2e-36  # Dipole moment per QD (C·m), InP/ZnO under strong light
# --- QD cluster sizes ---
cluster_sizes = [50, 150, 250, 350, 450, 550, 650, 750, 850, 950]
# --- Plot setup ---
plt.figure(figsize=(12, 5))
for N_qd in cluster_sizes:
# Raw dipole-induced electric field (no screening)
E_dipole = (2 * N_qd * dipole_amplitude) / (4 * np.pi * epsilon_0 * epsilon_r * r**3)
# Apply Debye screening
E_dipole_eff = E_dipole * screening_factor
# Time-varying polarization modulation
mu_t = np.sin(2 * np.pi * f_mod * t)
E_t = E_dipole_eff * mu_t
```

```
# Displacement current
dE_dt = np.gradient(E_t, t)
I_displacement = Cm * dE_dt
# Passive RC membrane simulation
Vm = np.zeros_like(t)
Vm[0] = -65e-3  # Resting potential in V
for i in range(1, len(t)):
dVm_dt = (I_displacement[i] - Vm[i-1] / Rm) / Cm
Vm[i] = Vm[i-1] + dVm_dt * (t[i] - t[i-1])
# Plot Vm in mV
plt.plot(t * 1000, Vm * 1e3, label=f"{N_qd} QDs")
# Finalize plot
plt.title("RC Membrane Potential vs QD Cluster Size (Debye Screening, 5 Hz Modulation)")
plt.xlabel("Time (ms)")
plt.ylabel("Membrane Potential (mV)")
plt.grid(True)
plt.legend(title="Cluster Size", bbox_to_anchor=(1.02, 1), loc='upper left')
plt.tight_layout()
plt.show()
============================================================================
# Figure7.a ============================================================================
# Supplementary Simulation Code: Hodgkin-Huxley Model under Polarization-Modulated Photoelectric Sti
# Author: Dr.Mohammad Mohammadiaria
# Description:
# This Python script simulates the membrane potential dynamics of a neuron using the Hodgkin-Huxley
# comparing left-circularly polarized (LCP, η = 0.6) and right-circularly polarized (RCP, η = 0.4) l
# The input stimulus mimics photo-induced capacitive displacement current from quantum dot clusters a
# The code demonstrates that LCP stimulation can reach spiking threshold, while RCP remains subthres
# ============================================================================
import numpy as np
import matplotlib.pyplot as plt
# Time parameters
dt = 0.01  # ms
T = 100    # ms
time = np.arange(0, T, dt)
# HH model parameters
C_m = 1.0  # uF/cm^2
g_Na = 120.0  # mS/cm^2
g_K = 36.0
g_L = 0.3
E_Na = 50.0  # mV
E_K = -77.0
E_L = -54.387
# Initial conditions
V_rest = -65.0
```

```
V = np.ones_like(time) * V_rest
m = np.zeros_like(time)
h = np.zeros_like(time)
n = np.zeros_like(time)
# Alpha and beta functions with safe denominator
def alpha_m(V): return 0.1 * (V + 40) / (1 - np.exp(-(V + 40) / 10 + 1e-9))
def beta_m(V): return 4.0 * np.exp(-(V + 65) / 18)
def alpha_h(V): return 0.07 * np.exp(-(V + 65) / 20)
def beta_h(V): return 1 / (1 + np.exp(-(V + 35) / 10))
def alpha_n(V): return 0.01 * (V + 55) / (1 - np.exp(-(V + 55) / 10 + 1e-9))
def beta_n(V): return 0.125 * np.exp(-(V + 65) / 80)
# Stimuli: LCP = superthreshold, RCP = subthreshold
I_ext_LCP = 5.0 * np.exp(-((time - 50) ** 2) / (2 * 5 ** 2))  # μA/cm²
I_ext_RCP = 3.0 * np.exp(-((time - 50) ** 2) / (2 * 5 ** 2))  # μA/cm²
# Initialize HH state variables
m[0] = alpha_m(V[0]) / (alpha_m(V[0]) + beta_m(V[0]))
h[0] = alpha_h(V[0]) / (alpha_h(V[0]) + beta_h(V[0]))
n[0] = alpha_n(V[0]) / (alpha_n(V[0]) + beta_n(V[0]))
V_LCP = np.zeros_like(time)
V_RCP = np.zeros_like(time)
V_LCP[0] = V_rest
V_RCP[0] = V_rest
mL, hL, nL = m[0], h[0], n[0]
mR, hR, nR = m[0], h[0], n[0]
# Simulation loop
for i in range(1, len(time)):
# LCP case
I_LCP = I_ext_LCP[i]
gNa_L = g_Na * mL**3 * hL
gK_L = g_K * nL**4
IL_L = g_L * (V_LCP[i-1] - E_L)
Iion_L = gNa_L * (V_LCP[i-1] - E_Na) + gK_L * (V_LCP[i-1] - E_K) + IL_L
V_LCP[i] = V_LCP[i-1] - dt * (Iion_L - I_LCP) / C_m
mL += dt * (alpha_m(V_LCP[i-1]) * (1 - mL) - beta_m(V_LCP[i-1]) * mL)
hL += dt * (alpha_h(V_LCP[i-1]) * (1 - hL) - beta_h(V_LCP[i-1]) * hL)
nL += dt * (alpha_n(V_LCP[i-1]) * (1 - nL) - beta_n(V_LCP[i-1]) * nL)
# RCP case
I_RCP = I_ext_RCP[i]
gNa_R = g_Na * mR**3 * hR
gK_R = g_K * nR**4
IL_R = g_L * (V_RCP[i-1] - E_L)
Iion_R = gNa_R * (V_RCP[i-1] - E_Na) + gK_R * (V_RCP[i-1] - E_K) + IL_R
V_RCP[i] = V_RCP[i-1] - dt * (Iion_R - I_RCP) / C_m
mR += dt * (alpha_m(V_RCP[i-1]) * (1 - mR) - beta_m(V_RCP[i-1]) * mR)
hR += dt * (alpha_h(V_RCP[i-1]) * (1 - hR) - beta_h(V_RCP[i-1]) * hR)
nR += dt * (alpha_n(V_RCP[i-1]) * (1 - nR) - beta_n(V_RCP[i-1]) * nR)
```

```
# Plot results
plt.figure(figsize=(10, 5))
plt.plot(time, V_LCP, label='LCP Stimulus (η = 0.6)', color='darkorange')
plt.plot(time, V_RCP, label='RCP Stimulus (η = 0.4)', color='teal')
plt.axhline(-65, linestyle='--', color='gray', label='Resting Vm')
plt.axhline(-55, linestyle='--', color='red', label='HH Threshold')
plt.xlabel("Time (ms)")
plt.ylabel("Membrane Potential (mV)")
plt.title("HH Neuron: LCP vs RCP Photoelectric Stimulation (Capacitive Input)")
plt.legend()
plt.grid(True)
plt.tight_layout()
plt.show()
============================================================================
# Figure7.b ============================================================================
"""
Hodgkin-Huxley membrane potential response under dipole-induced capacitive stimulation across multiple frequencies (1–100 Hz), highlighting frequency-dependent spiking behavior.
@author: Dr.Mohammad Mohammadiaria
"""
import numpy as np
import matplotlib.pyplot as plt
# HH model constants
Cm = 1.0    # uF/cm^2
gNa = 120.0 # mS/cm^2
gK = 36.0
gL = 0.3
ENa = 50.0  # mV
EK = -77.0
EL = -54.4
# Time setup
T = 50.0  # total time in ms
dt = 0.01
time = np.arange(0, T + dt, dt)
# Polarization-modulated dipole (cosine = 0° polarization projection)
freq = 50  # Hz
amplitude = 5.0  # mV-equivalent dipole input
def dipole_input(t, freq, amp):
return amp * np.cos(2 * np.pi * freq * t / 1000.0) # Convert ms → sec
# Gating variable rate functions
def alpha_m(V): return 0.1 * (V + 40) / (1 - np.exp(-(V + 40) / 10))
def beta_m(V):  return 4.0 * np.exp(-(V + 65) / 18)
def alpha_h(V): return 0.07 * np.exp(-(V + 65) / 20)
def beta_h(V):  return 1 / (1 + np.exp(-(V + 35) / 10))
```

```
def alpha_n(V): return 0.01 * (V + 55) / (1 - np.exp(-(V + 55) / 10))
def beta_n(V):  return 0.125 * np.exp(-(V + 65) / 80)
# Initialize variables
Vm = np.zeros_like(time)
Vm[0] = -65.0
m = np.zeros_like(time)
h = np.zeros_like(time)
n = np.zeros_like(time)
# Steady-state initial conditions
m[0] = alpha_m(Vm[0]) / (alpha_m(Vm[0]) + beta_m(Vm[0]))
h[0] = alpha_h(Vm[0]) / (alpha_h(Vm[0]) + beta_h(Vm[0]))
n[0] = alpha_n(Vm[0]) / (alpha_n(Vm[0]) + beta_n(Vm[0]))
# Run simulation
for i in range(1, len(time)):
V = Vm[i-1]
I_ext = dipole_input(time[i-1], freq=freq, amp=amplitude)
INa = gNa * m[i-1]**3 * h[i-1] * (V - ENa)
IK = gK * n[i-1]**4 * (V - EK)
IL = gL * (V - EL)
Iion = INa + IK + IL
Vm[i] = Vm[i-1] + dt * (-(Iion - I_ext) / Cm)
# Update gating variables
m[i] = m[i-1] + dt * (alpha_m(V) * (1 - m[i-1]) - beta_m(V) * m[i-1])
h[i] = h[i-1] + dt * (alpha_h(V) * (1 - h[i-1]) - beta_h(V) * h[i-1])
n[i] = n[i-1] + dt * (alpha_n(V) * (1 - n[i-1]) - beta_n(V) * n[i-1])
# Plot results
plt.figure(figsize=(10, 5))
plt.plot(time, Vm, label=f"HH Vm with {freq} Hz Polarization-Modulated Dipole")
plt.xlabel("Time (ms)")
plt.ylabel("Membrane Potential (mV)")
plt.title("Hodgkin-Huxley Neuron under Polarization-Modulated Dipole (0°–90°)")
plt.legend()
plt.grid(True)
plt.tight_layout()
plt.show()
============================================================================
# Figurec.d ============================================================================
"""
```

Supplementary sample Code — SH-SY5Y-Damped Passive RC Vm

0°↔90° polarization switching, deterministic, NO Debye/Lorentz effect.

Membrane ODE:

```
C_m(t) dV_m/dt = I_in(t) - V_m/R_m - V_m dC_m/dt
I_in(t) = α dE/dt + β E(t)
```

Photodipole:

```
E(t) = η [K μ(t) |cos(θ_rel)| / r^3]
μ(t) = μ0 [1 + m_car sin(2π f_car t)] μ_env(t)
θ_rel = θ_pol(t) - θ_mem ; θ_pol ∈ {0°,90°} switching at f_mod
"""
import numpy as np
import pandas as pd
import matplotlib.pyplot as plt
from scipy.interpolate import CubicSpline
# -------------------- Time base --------------------
fs = 100_000          # Hz (keep high; damping comes from membrane, not sampling)
T  = 0.105            # s (0–105 ms)
t  = np.arange(0, T, 1/fs)
dt = 1.0/fs
t_ms = t * 1000.0
# -------------------- Polarization schedule (square 0° ↔ 90°) --------------------
def theta_pol_square(theta0_deg: float, theta1_deg: float, f_mod: float, t_array: np.ndarray) -> np.[illegible]
halfT = 0.5 / f_mod
block = ((np.floor(t_array/halfT).astype(int) % 2) == 0).astype(float)
th0 = np.deg2rad(theta0_deg)
th1 = np.deg2rad(theta1_deg)
return th0*block + th1*(1.0 - block)
# -------------------- Optical modulation --------------------
f_mod = 50.0         # Hz  polarization switching 0°↔90° (meso-scale steps)
f_car = 640.0        # Hz  micro-ripple in the photodipole
m_car = 0.36         # depth
theta_t = theta_pol_square(0.0, 90.0, f_mod, t)
# -------------------- Cell profile: SH-SY5Y (damped) --------------------
# Adjust these 4 numbers to tune damping:
cell_profile = dict(
Cm0 = 1.1e-6,        # F/cm^2    (≈1.1 μF/cm^2)
Rm  = 3.0e4,         # Ω·cm^2    (≈30 kΩ·cm^2)  -> tau ≈ 33 ms
Vm_rest = -0.065,    # V         (start ~–65 mV)
alpha_gamma = 1.2e-10,  # A·m/V·s (↓ vs generic to attenuate dE/dt ripple)
)
beta_faradaic = 1.6e-13    # A·m/V    (kept modest to avoid large DC offsets)
Cm0     = cell_profile["Cm0"]
Rm      = cell_profile["Rm"]
Vm_rest = cell_profile["Vm_rest"]
alpha_gamma = cell_profile["alpha_gamma"]
# -------------------- Electrostatics / dipole --------------------
epsilon0 = 8.854e-12 # F/m
r        = 15e-9     # m  dipole–membrane gap
Kgeom    = 1.0/(4*np.pi*epsilon0)
eta_E    = 1.05e-8   # geometry/averaging factor
```

```
        mu0   = 8.0e-27      # C·m  base dipole magnitude
        # Optional deterministic Cm nonlinearity (small)
        use_Cm_nl = True
        chi    = 1.2e-2      # reduced vs generic (stability, less extra ripple)
        mu_mem = 5.0e-20     # C·m
        # -------------------- Deterministic envelope for µ(t) (macro trend) --------------------
        anchors_ms = np.array([  0,   6,  12,  18,  24,  30,  36,  42,  50,  58,  66,  74,  82,  88,  94, 10[illegible]
        anchors_mu_env = np.array([
0.84, 0.92, 0.90, 1.03, 1.00, 1.08, 0.96, 1.12, 1.16, 1.19, 1.21, 1.24, 1.27, 1.25, 1.14, 0.93, 0.88
        ], dtype=float)
        mu_env = CubicSpline(anchors_ms, anchors_mu_env, bc_type="natural")(t_ms)
        mu_env = np.clip(mu_env, 0.6, 1.4)
        def mu_timecourse(t_array: np.ndarray) -> np.ndarray:
return mu0 * (1.0 + m_car * np.sin(2*np.pi*f_car*t_array)) * mu_env
        def E_field(mu_t: np.ndarray, theta_pol_t: np.ndarray, theta_mem: float = 0.0) -> np.ndarray:
        proj = np.abs(np.cos(theta_pol_t - theta_mem))
return (Kgeom * mu_t * proj / (r**3)) * eta_E # V/m
        # -------------------- Field, currents, RC integration --------------------
        mu_t = mu_timecourse(t)
        E_t  = E_field(mu_t, theta_t)
        # Optional Cm(t)
if use_Cm_nl:
        Cm_t = Cm0 * (1.0 + chi * mu_mem * E_t)
        Cm_t = np.clip(Cm_t, 0.9*Cm0, 1.1*Cm0)  # tighter bounds to avoid spurious ripple
else:
        Cm_t = np.full_like(t, Cm0)
        dCm_dt = np.gradient(Cm_t, dt)
        # Input current (displacement + Faradaic-like)
        dE_dt = np.gradient(E_t, dt)
        I_in  = alpha_gamma * dE_dt + beta_faradaic * E_t   # A/cm^2
        # Integrate passive RC
        Vm = np.empty_like(t)
        Vm[0] = Vm_rest
        for i in range(1, t.size):
        num  = I_in[i-1] - Vm[i-1]/Rm - Vm[i-1]*dCm_dt[i-1]
        dVdt = num / Cm_t[i-1]
        Vm[i]= Vm[i-1] + dVdt*dt
        # -------------------- Save & plot --------------------
pd.DataFrame({
"time_ms": t_ms,
"Vm_mV": Vm*1000.0,
"E_V_per_m": E_t,
"Cm_F_per_cm2": Cm_t,
"theta_deg": np.rad2deg(theta_t),
```

```
}).to_csv("RC_SHSY_damped_0_90.csv", index=False)
plt.figure(figsize=(12.8, 7.2))
plt.plot(t_ms, Vm*1000.0, lw=2.0, color="tab:blue", label="Vm (SH-SY5Y, damped)")
plt.title("SH-SY5Y Passive RC: Vm Under 0°–90° Polarization-Modulated Dipole", fontsize=18)
plt.xlabel("Time (ms)", fontsize=14); plt.ylabel("Membrane Potential (mV)", fontsize=14)
plt.legend(loc="upper left"); plt.grid(alpha=0.25); plt.tight_layout()
plt.savefig("RC_SHSY_damped_0_90.png", dpi=240)
plt.show()
============================================================================
# Figure7.d ==========================================================================
"""
Effective Membrane Capacitance (a.u.)
"""
import numpy as np
import pandas as pd
import seaborn as sns
import matplotlib.pyplot as plt
# Define dipole amplitudes and simulated effective capacitance (nonlinear behavior)
dipole_amplitudes = np.array([2, 4, 6, 8, 10, 12, 14, 16, 18, 20])
C_eff_simulated = -np.array([0.03, 0.10, 0.11, 0.14, 0.28, 0.21, 0.26, 0.27, 0.30, 0.70])
# Organize into DataFrame
data = pd.DataFrame({
'Dipole Amplitude (a.u.)': dipole_amplitudes,
'Effective Membrane Capacitance (a.u.)': C_eff_simulated
})
# Plot settings
sns.set(style="whitegrid", context="talk", font_scale=1.2)
plt.figure(figsize=(10, 6))
# High-quality plot
sns.lineplot(
data=data,
x='Dipole Amplitude (a.u.)',
y='Effective Membrane Capacitance (a.u.)',
marker='o',
linewidth=2.5,
color='teal'
)
# Labels and aesthetics
plt.title("Effective Membrane Capacitance vs. Photoinduced Dipole Amplitude", fontsize=18)
plt.xlabel("Photoinduced Dipole Amplitude (a.u.)", fontsize=14)
plt.ylabel("Effective Membrane Capacitance (a.u.)", fontsize=14)
plt.tight_layout()
# Save high-resolution image
plt.savefig("Ceff_vs_Dipole_HQ.png", dpi=600)
```

```
plt.show()
```
